# Distinct distributions of genomic features of the 5’ and 3’ partners of coding somatic cancer gene fusions: arising mechanisms and functional implications

**DOI:** 10.18632/oncotarget.10734

**Published:** 2016-07-20

**Authors:** Yongzhong Zhao, Won-Min Song, Fan Zhang, Ming-Ming Zhou, Weijia Zhang, Martin J. Walsh, Bin Zhang

**Affiliations:** ^1^ Department of Genetics and Genomic Sciences, Icahn School of Medicine at Mount Sinai, NY 10029, USA; ^2^ Institute of Genomics and Multiscale Biology, Icahn School of Medicine at Mount Sinai, NY 10029, USA; ^3^ Department of Medicine, Icahn School of Medicine at Mount Sinai, NY 10029, USA; ^4^ Department of Structural and Chemical Biology, Icahn School of Medicine at Mount Sinai, NY 10029, USA; ^5^ Department of Pediatrics, Icahn School of Medicine at Mount Sinai, New York, NY 10029, USA

**Keywords:** cancer somatic gene fusions, gene age, GC skew, DNA-RNA R-loops, somatic amplification

## Abstract

The genomic features and arising mechanisms of coding cancer somatic gene fusions (CSGFs) largely remain elusive. In this study, we show the gene origin stratification pattern of CSGF partners that fusion partners in human cancers are significantly enriched for genes with the gene age of*Euteleostomes* and with the gene family age of *Bilateria*. GC skew (a measurement of G, C nucleotide content bias, (G-C)/(G+C)) is a useful measurement to indicate the DNA leading strand, lagging strand, replication origin, and replication terminal and DNA-RNA R-loop formation. We find that GC skew bias at the 5 prime (5′) but not the 3 prime (3’) partners of CSGFs, coincident with the polarity feature of gene expression breadth that the 5’ partners are more ubiquitous while the 3’ fusion partners are more tissue specific in general. We reveal distinct length and composition distributions of 5’ and 3’ of CSGFs, including sequence features corresponded to the 5’ untranslated regions (UTRs), 3’ UTRs, and the N-terminal sequences of the encoded proteins. Oncogenic somatic gene fusions are most enriched for the 5’ and 3’ genes’ somatic amplification alongside a substantial proportion of other types of combinations. At the function level, 5’ partners of CSGFs appear more likely to be tumour suppressor genes while many 3’ partners appear to be proto-oncogene. Such distinct polarities of CSGFs at the evolutionary, structural, genomic and functional levels indicate the heterogeneous arsing mechanisms of CSGFs including R-loops and suggest potential novel targeted therapeutics specific to CSGF functional categories.

## INTRODUCTION

Somatic gene fusions are a common feature of many human cancers and have been found prevalent in leukemia, sarcoma, as well as epithelial cancers such as thyroid, prostate, colorectal, and breast cancer [1, 2]. CSGF data have been well-curated and publically available through the Mitelman Database of Chromosome Aberrations and Gene Fusions in cancer [2], the COSMIC database [3], the TICdb [4], as well as in publications [5–7]. Genome-wide studies of CSGFs, especially in hematological malignant diseases, have considered the involvement of chromatin structure [8] and timing during replication [9] as putative genomic susceptibilities for the arising of CSGFs. Recently, CSGFs have been systematically identified in tumor samples of The Cancer Genome Atlas (TCGA) project [6] and in cancer cell lines [5]. These highly heterogeneous data have been offering an unprecedented opportunity to study the arising mechanisms and function of CSGFs.

Cancers evolve by a reiterative somatic process of clonal expansion, genetic diversification and clonal selection within the adaptive landscapes of tissue ecosystems. [10, 11], relying on the somatic dynamics of genomic features and functions. Individual oncogenic fusions can also have inherent tumor suppression property [12]. Meanwhile, the natural history of genes links to diseases, especially for cancers [13, 14]. It appears that arsing mechanisms and genomic functions of CSGFs are highly heterogeneous [15–21]. Meanwhile, little known is whether there are distinct phylogenomic and genomic features of CSGFs involved genes. In this study, we sought to link the phylogenomic and genomic features of CSGFs involved genes to potential arsing mechanisms and function categories of CSGFs by leveraging the rich resources of omics data.

## RESULTS

### CSGF data sets and annotation settings

We collected six curated datasets of CSGFs (Table 1), including the COSMIC [22], the cancer cell line fusion data (Genetech) [5], TICdb [4], the ensemble cancer landscape fusion data [7], Mitelman fusion data (Mitelman) [23], and the TCGA fusion data (TCGA) [6]. Toward a comprehensive annotation of CSGFs involved genes, we also collected a number of functional genomic data sets (Table 1), including the reference human genomic ENSEMBL data [24], protein-protein interactions [25], the FANTOM expression data [26, 27], GTEX data [28], the standard gene expression data set [29], Illumina's body map [30], the draft human proteome [31, 32], the gene and gene family age curation [14], the human gene expression atlas [33], as well as the TCGA data [34–36]. Of note, these data sets have been well recognized for their quality. Many are standard setting yet highly heterogeneous, raising substantial challenge for signals capturing in terms of the following analysis. Thus, if significant signal captured, it may have much implications.

**Table 1:** List of CSGF data and annotation settings

Data description	Abbreviation	PMID
the COSMIC database	COC	25355519
the TCGA fusion data	CLF	25485619
the database of translocation breakpoints in cancer	TIC	17257420
the curated cancer landscape gene fusions	BKF	23539594
The Mitelman gene fusion database	MIF	17361217
the gene fusion in cancer cell lines	TCF	25500544
Su AI expression data 2004	SAIe	15075390
the curated protein coding	CPCh	24939910
the draft human proteome	DHPe	24870542
protein-protein interaction network	PPIn	25416956
The Cancer Genome Atlas	TCGA	25109877
Ensembl	ENSe	25352552
Fantom5	FANt	24670764
tissue-expression map	TEMe	25613900
Illumina Body map	IBMe	24217909
GTEX expression	GTEx	25954003
gene age curated	GACu	20492640
human gene expression atlas	HGEa	20379172
somatic mutation rate and replication time	SMRr	23770567

These data include six gene fusion sets and thirteen annotation data settings.

### Genome wide preference pattern of genes as 5′ partner (5′FG) or 3′ partner (3′FG) of CSGFs

**Figure 1:**
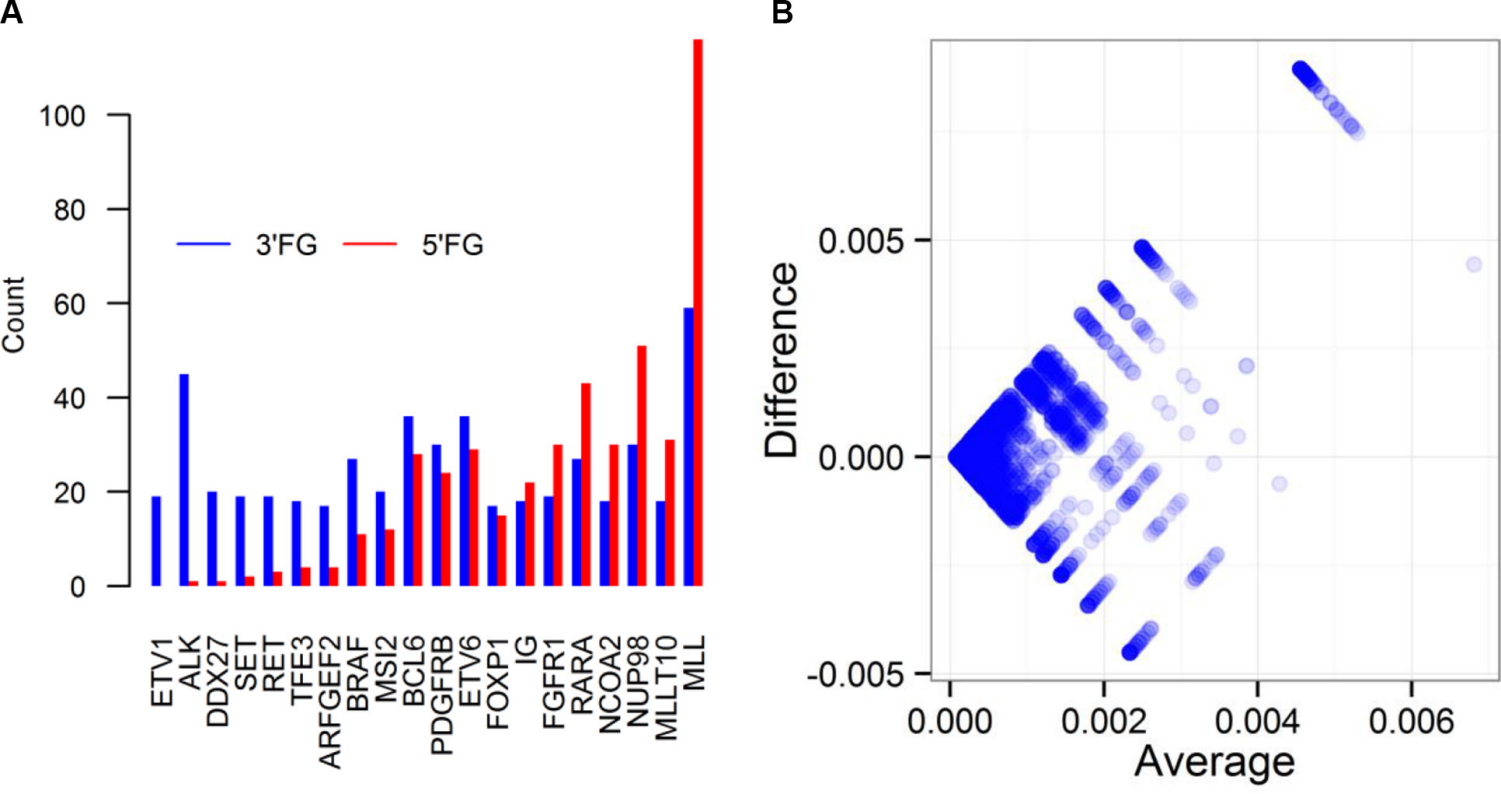
Genome wide preference pattern of genes as 5′ partner (5’FG) or 3′ partner (3’FG) of CSGFs (**A)** The distributions of the top 20 frequent CSGFs involved genes in terms of preference of 5’FG or 3’FG in the fusion gene sets. (**B)** The distribution of CSGFs involved genes is illustrated with the Bland-Altman plot (*n* = 12848, Kolmogorov-Smirnov test D = 0.54, *p*-value < 2.2e-16). The x-axis (average) indicates the average frequency of each involved gene in terms of 5′ and 3′, while the y-axis (difference) indicates the difference between 5′ and 3′ frequency of each gene.

Little is known about the genome wide preference pattern of being 5′FG or 3′FG of CSGFs. Of the 12,849 unique CSGFs collected from the resources listed in Table 1, we found 5,980 genes at 5′ (or N terminal at the protein coding level) and 7,150 genes at 3′ (or C-terminal at the protein coding level), as well as 3,357 genes observed at both directions. The top 20 most frequent CSGFs involved genes have distinct preferences of being involved in 5′FG or 3′FG (Figure 1A). For examples, *ALK* is frequently fused at the 3′ end while *RARA* is more frequently fused at the 5′ end (Figure 1A). The genome-wide distribution is shown in the Bland-Altman plot, illustrating the global asymmetry (*n* = 12,848, Kolmogorov-Smirnov test D = 0.54, *p*-value < 2.2e-16) (Figure 1B). Thus, it appears that being as 5′FG or 3′FG is highly asymmetric at both the global and individual gene levels, indicating a potential link of gene features to the arising of CSGFs.

### *Euteleostomes* genes and *Bilateria* gene families are more likely to be involved in CSGFs

**Figure 2:**
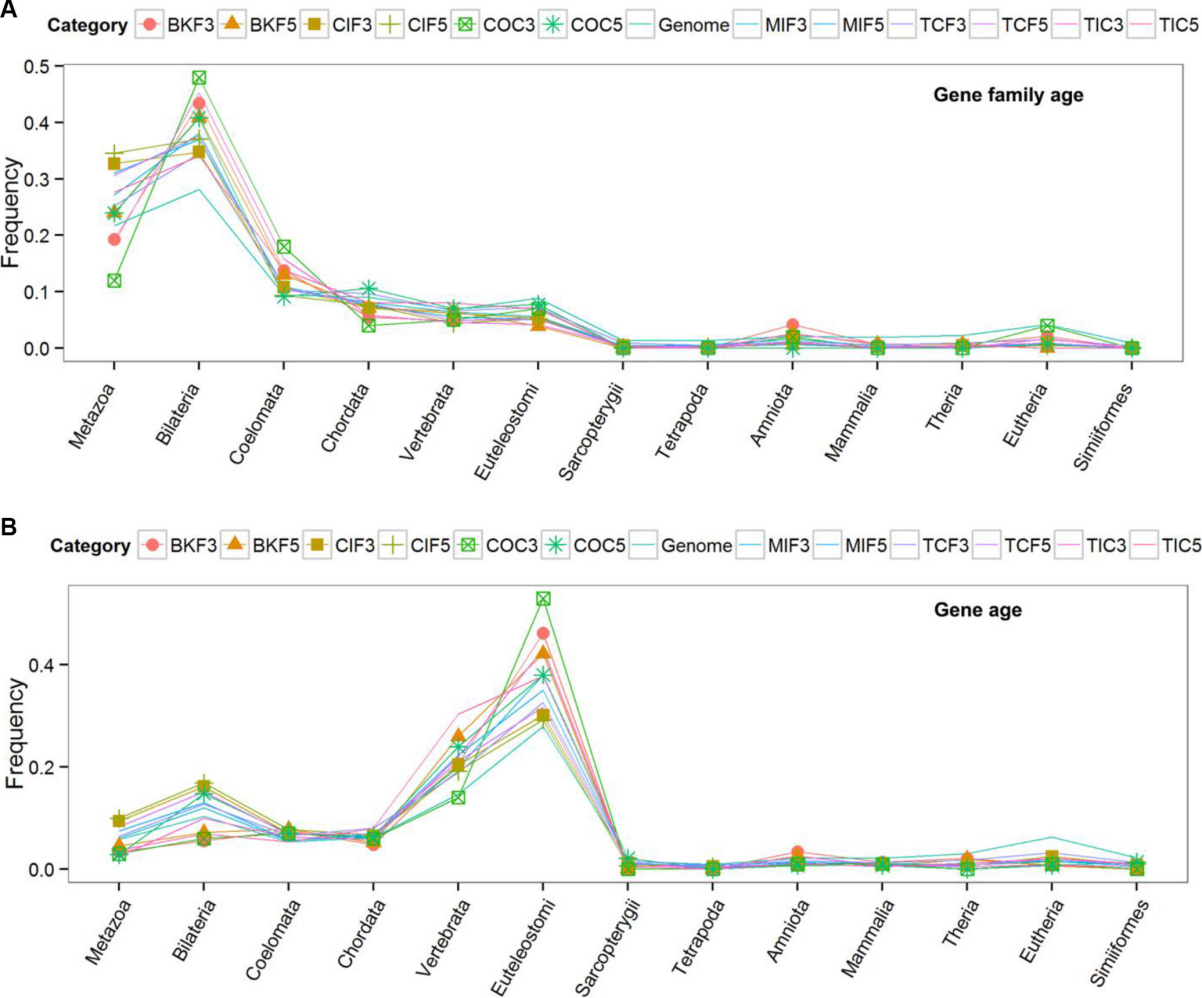
Phylogenomic profiles of cancer fusion involved genes The gene family ages (upper) and the gene ages (lower) of the CSGF involved genes. The x-axis shows the distinct phylogenetic ages with corresponded the birth of distinct species. The y-axis indicates the proportion of each stratified phylogenetic ages. The abbreviation of each category is described to Table 1 while the ‘5’ or ‘3’ postfix indicates the 5′ or 3′ involved genes respectively. The “Genome” category represents the average level of genes of the human genome.

**Figure 3:**
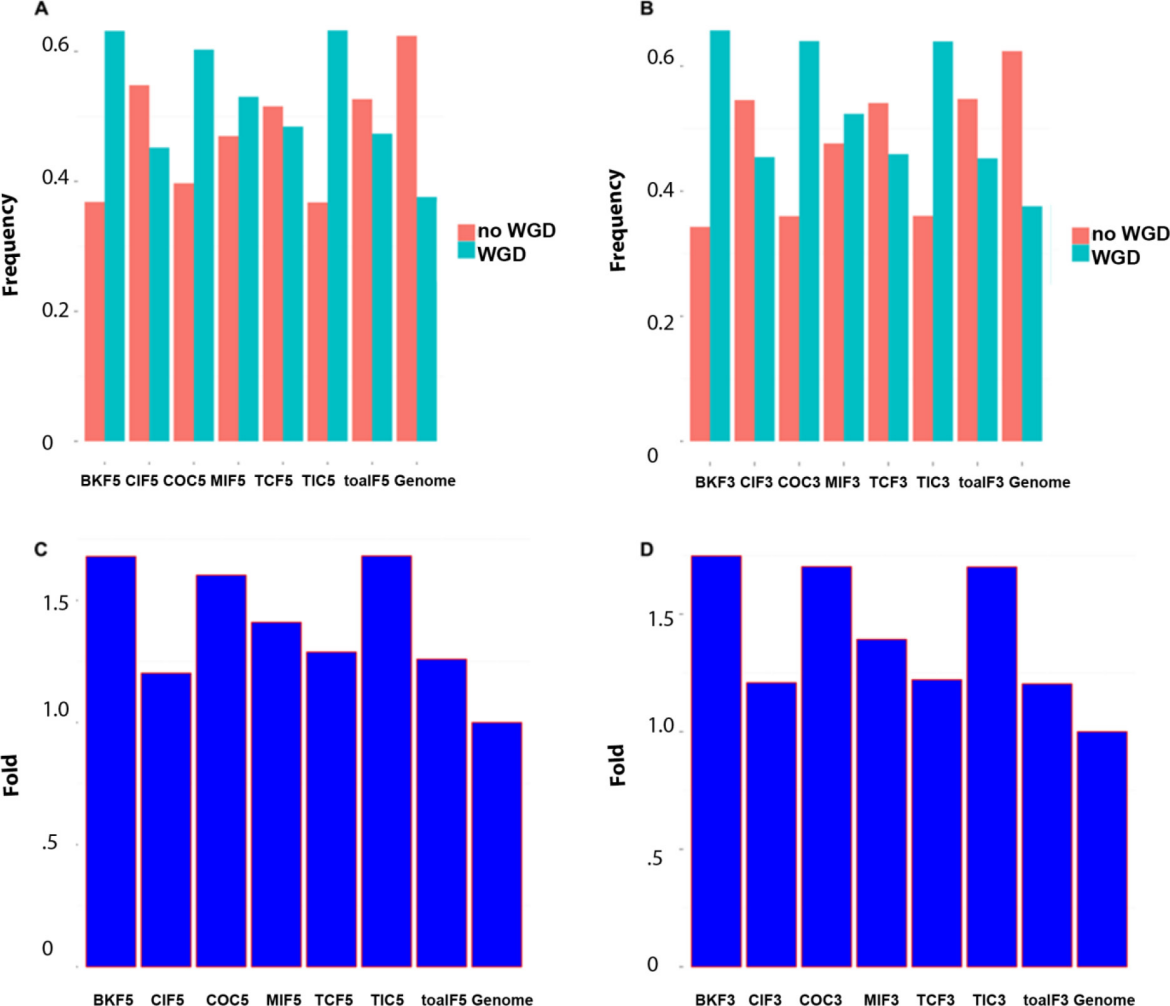
CSGFs involved genes are more related to whole genome duplications (WGD) **(A)** The proportion of gene origin involved in the WGD event. **(B)** The proportion of gene origin not involved in the WGD event. **(C)** Fold enrichment of gene origin involved in the WGD event. **(D)** Fold enrichment involved in whole genome duplication. The abbreviation of each category is described to Table 1 while the ‘5’ or ‘3’ postfix indicates the 5′ or 3′ involved genes respectively.

**Figure 4:**
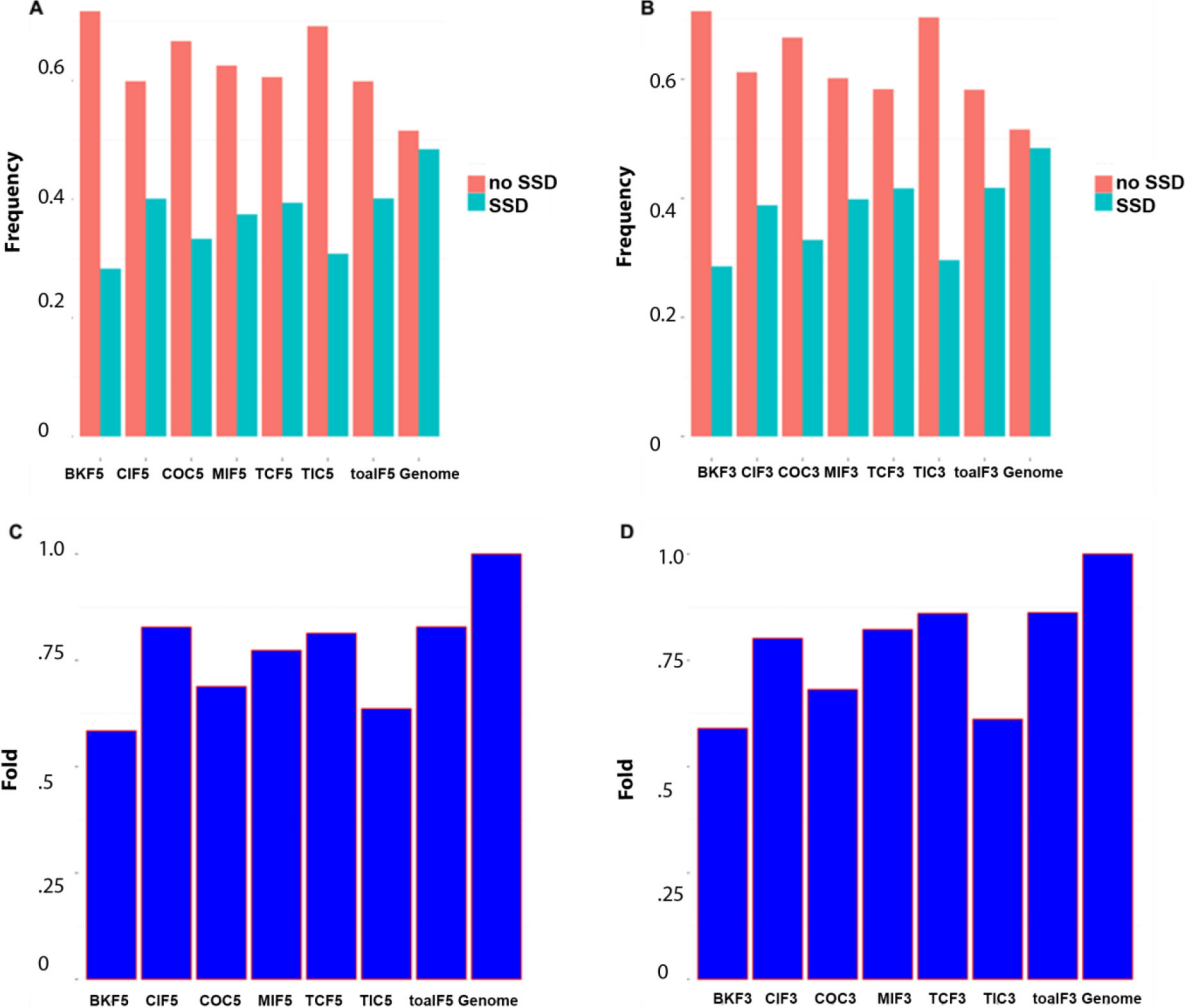
CSGFs involved genes are under-presented with small scale genome duplications (**A, B**) The proportion of genes origin involved the small scale genome duplication event and non-involved. (**C, D**) Fold enrichment involved in small scale duplication. The abbreviation of each category is described to Table 1 while the ‘5’ or ‘3’ postfix indicates the 5′ or 3′ involved genes respectively.

To exploit phylogenomic profile of CSGFs involved genes, we collected three phylogenomic profile data sets, including the gene age database Protein historian [37], as well as two additional curated data sets [14, 38]. As shown Table 2, the origins of most recurrent fusions involved genes are almost of a vertebrate origin. CSGF involved genes are significantly enriched of the *Bilateria* origin in terms of gene family (Figure 2A) and *Euteleostomes* origin (Figure 2B) of gene age. The 3′ portion of driver CSGFs set (COC3, from the COSMIC data set) is among the highest enriched set of genes. Consistently, CSGFs involved genes are more related to whole genome duplication events (Figure 3), but not small scale genome duplication events (Figure 4). These phylogenomic data appear to support a view that cancer is more likely a vertebrate-specific disease [39].

**Table 2:** Origins of most recurrent fusions involved genes

vertebrate specific domain combination	vertebrate specific duplication	metazoan specific domain
SLC34A2	BCL6	RARA
MLL	CHD7	ERG
RET	MLL	
FGFR1	IGH2	
ABL1	ETV6	
MYC	NUP98	
EML4	RUNX1	
BCR	PAX5	
PML	EWSR1	
TMPRSS2	TRB2	
MAGI3	IGL2	
PVT1	HMGA2	
MLL	PDGFRB	
FGFR1	ALK	
ROS1	ETV1	
ABL1	RSPO2	
MYC	ALK	
NDRG1	AKT3	
AF4	RSPO3	
TACC1		

### The asymmetric sequence pattern of CSGFs involved genes

Notably, there are at least four mechanisms by which how translocations arise: synthesis mediated end joining, breakage-fusion-bridge cycles, RAG-mediated translocation and AID-mediated translocation [40]. To gain insights into the arising mechanisms of CSGFs, we carried out global analysis of genomic sequence features of CSGFs involved genes, including the GC content defined as (G+C)/(T+G+C+A), the GC skew defined as the ratio of (G−C)/(G+C), and the AT skew defined as the ratio of (A−T)/(A+T)), as well as the S defined as the summary of GC skew and AT skew. An emerging asymmetric pattern of these basic sequence measurements can be utilized for prediction of R-loop formation [41]. R loops are three-stranded nucleic acid structures comprised of nascent RNA hybridized with DNA template while leaving the non-template DNA single-stranded [42]. R loops are considered more unstable intermediates of RNA-DNA structure and are preferentially formed when the non-template strand is G rich [43].

**Figure 5:**
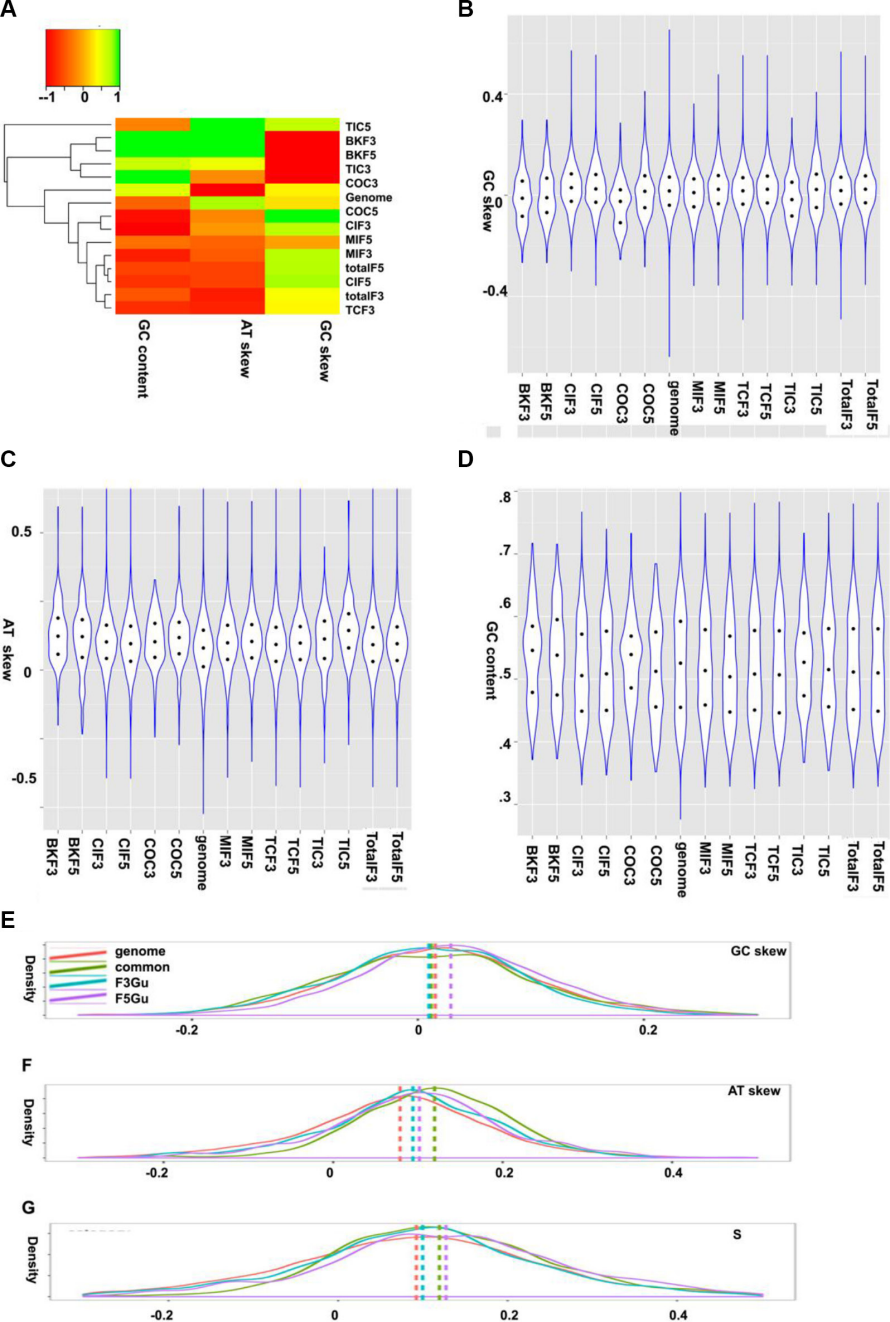
GC and AT skew features of CSGFs involved genes (**A, B, C,** and **D**) The GC and AT skew features of CSGF involved genes. GC skew, (G−C)/(G+C); AT skew, (T−A)/(A+T). Distinct strand asymmetric patterns of fusion genes are illustrated by Kernel density plotting of the three parameters for strand asymmetries including GC skew, AT skew and S composition. (**E**) The distribution patterns of GC-skew show the significant GC skew specific to the 5′ partner specific CSGFs involved genes (*P* < 2.2E-16). (**F**) The AT-skew variable distributions shows all fusion involved genes are more AT asymmetric compared to the background coding sequences (*P* < 2.2E-16). (**G**) A plot of the distribution of S composition measurement (S = GC_skew +AT_skew), show significant asymmetric distribution of CSFiGs (*P* < 2.2E-16). genome: the human genome, common: coding sequences involved as both 5′ partner and 3′ partner in the CSGF data sets, F3Gu: coding sequences involved as the 3′ partner, F5Gu: coding sequences involved as the 5′ partner. The vertical intersected lines indicate the median value of the variables of each category.

It appears to be AT rich in the 5′ partners of the CSGF driver set (COC, the COSMIC CSGF drivers) and GC rich in the 3′ partners (Figure 5A, scaled heatmap plot). Due to the highly heterogeneous nature of our collected data, the GC and AT skew features of CSGFs involved genes are highly heterogeneous (Figure 5B–5D). However, distinct strand asymmetric patterns of fusion genes were still observed (kernel density plot, Figure 5E) by using three parameters for measuring the strand asymmetries, including GC skew, AT skew and S composition. There is a significant GC skew towards the 5′ fusion partners (Figure 5E, *P* < 2.2e-16). Moreover, the AT-skew distribution shows that all fusion-involved genes are more AT asymmetric when compared to the background coding sequences (*P* < 2.2e- 16, Figure 5E). Consistently, the S composition shows a significant asymmetric distribution of CSGFs involved genes (*P* < 2.2e-16, Figure 5E).

**Figure 6:**
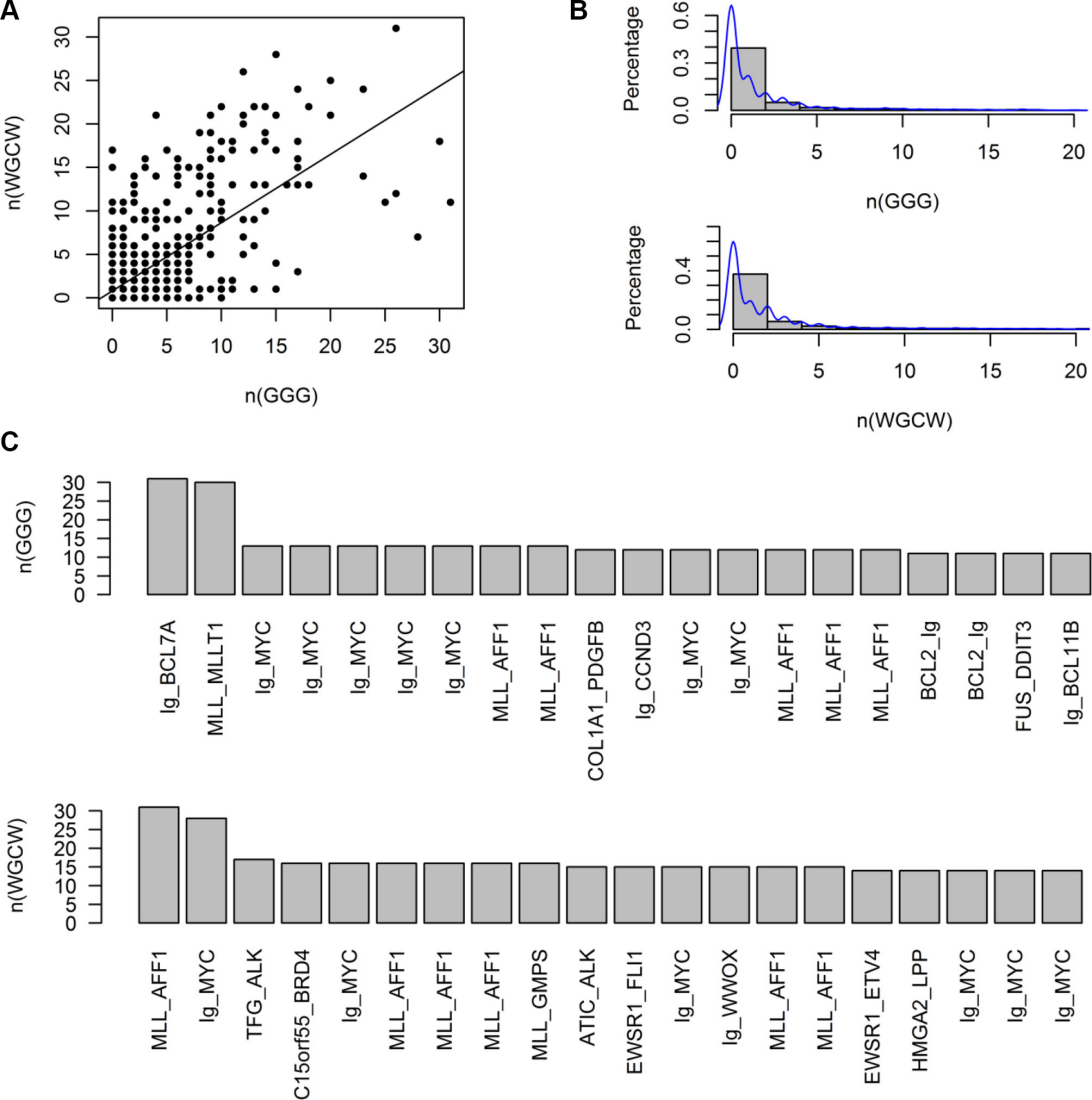
G-clusters, which initiate R loop formation, and on the number of WGCW sites, at a subset of CSGF breakpoint sequences The breakpoint sequences were downloaded from the TICdb (release 3.3: August 2013). (**A**) R-loop signature (GGG) is significantly correlated with IgH switch class recombination signature (WGCW) (Spearman correlation coefficient *ρ* = 0.5068, *p* = 1.57e-90). (**B**) The distribution of GGG and WGCW sites. (**C**) CSGFs are characterized by IgH class switch recombination signatures.

Since the DNA breakpoint sequence of CSGFs can reveal distinct translocation mechanisms, we set out to examine the motif patterns of known CSGFs breakpoints. The breakpoint sequences were downloaded from TICdb (Release 3.3, August 2013). Of note, we first analysis the global pattern of these breakpoints (Supplementary Figure S1), indicating an asymmetric pattern of the tetra-nucleotide distribution. Further, we performed detained key CSGFs analysis. As shown in Figure 6A, there is a direct correspondence between R-loop signature (GGG) with IgH class switch recombination signature (WGCW) (Spearman correlation coefficient *ρ* = 0.51, *p* = 1.57e-90). However, it is only a subset of CSGFs characterized by R-loops and an IgH switch signature (Figure 6B). Related CSGFs were listed, including Ig-MYC (Figure 6C). Thus, given these signals, our sequence analysis results support the potential of R-loops involved in arising mechanism in a subset of CSGFs.

### Distinct genomic features including gene expression breath of CSGFs involved in genes

**Figure 7:**
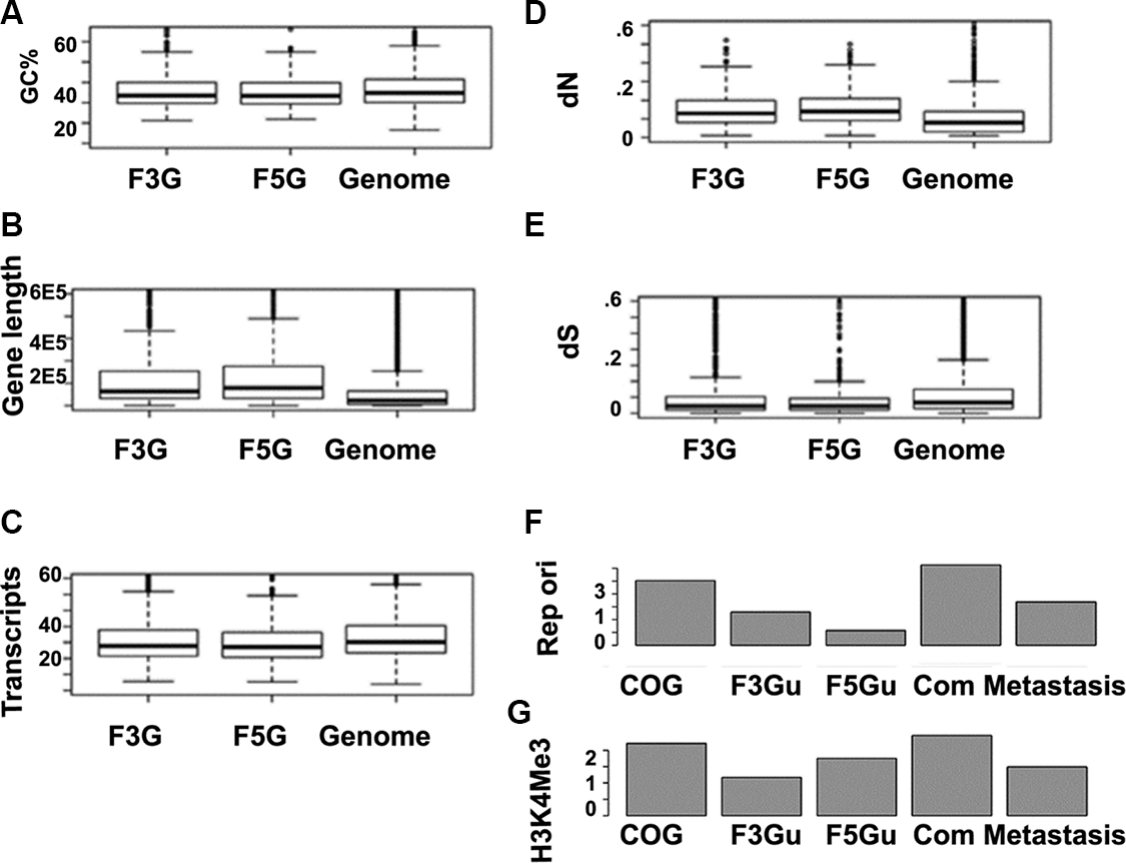
Illustrating distinct features of CSGF involved genes based on genome features (**A**) F3Gs and F5Gs appear to be highly GC rich. GC, (G+C)/(A+T+C+G) for the coding strand. (**B**) Longer gene length (from the transcription start site to the poly adenalyation signal site. (**C**) More transcripts, indicative of more alternative splicing forms. (**D**) Higher non-synonymous mutation rate (dN) for the comparison between human and mouse othologs.(**E**) Lower mutation rate represented with human-mouse synonymous changes (dS). (**F**) Distinct replication origin signatures of 5′ or 3′ cancer somatic involved gene are illustrated, (**G**) Gene activity markers H3K4Me3 enrichments of different gene sets, including cancer genes (COSMIC v72 cancer genes), F3Gu (CSGFs involved 3′specific genes), F5Gu (CSGFs 5′ involved specific genes), FGcommon (genes involved in 3′ and 5′ both), and Metastasis (metastasis gene set).

**Figure 8:**
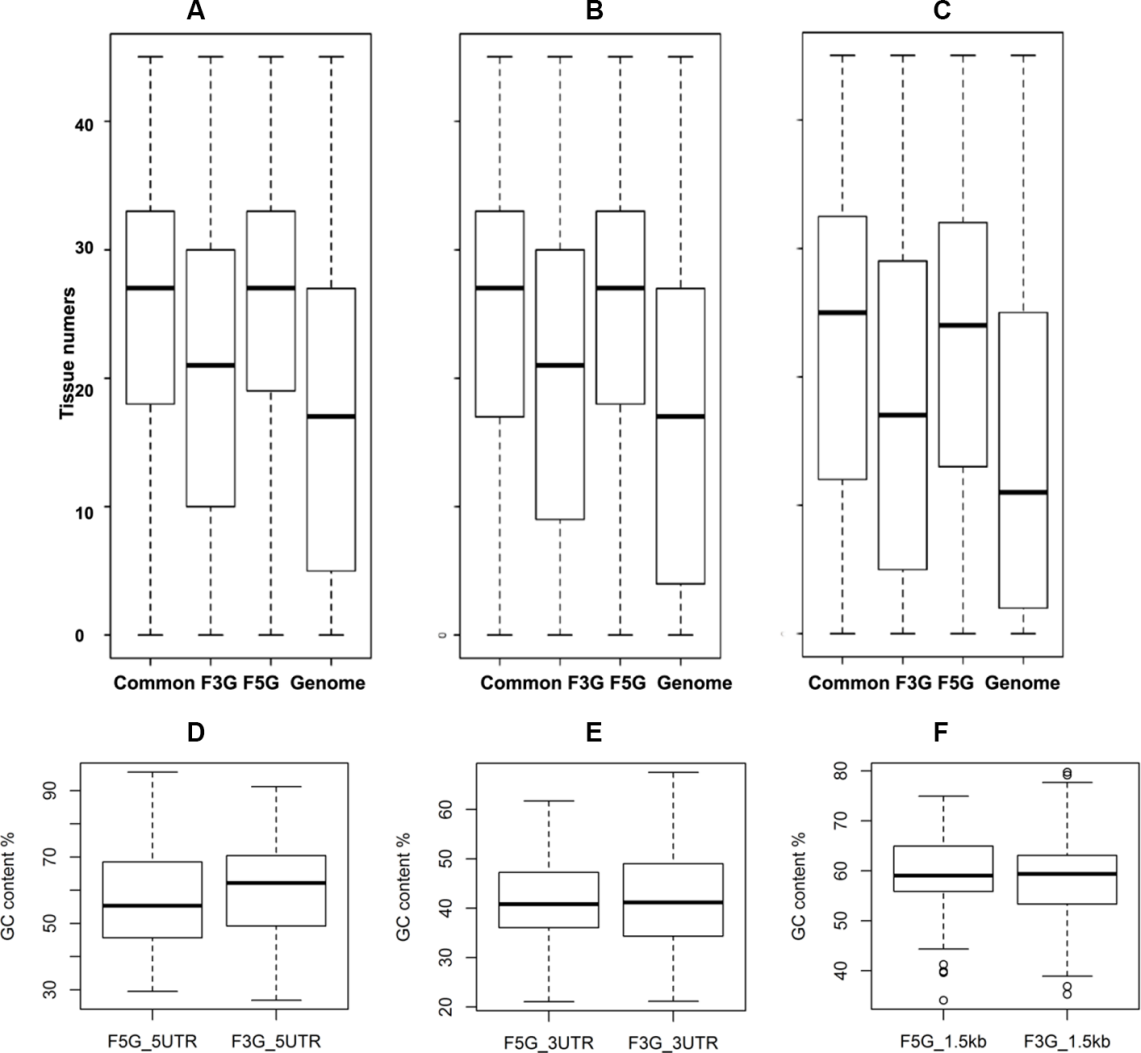
Asymmetric patterns of gene expression pattern and GC content of UTRs of cancer somatic gene fusion involved genes The expression of CSGFs involved genes show the polarity of CSGFs at the expression level. The y-axis shows tissue numbers in terms of gene expression with distinct threshold of reference RNA-seq data, including 5 (**A**), 10 (**B**), or 20 (**C**) FPKM (fragments per kilobase per million). (**D**) 5′UTR comparison, Wilcoxon rank sum test, W = 257,110, *P* = 1.95e-05. (**E**) 3′ UTR comparison, Wilcoxon rank sum test with continuity correction, W = 129,190, *P* = 0.53. (**F**) Promoter comparison, Wilcoxon rank sum test, W = 18896, *P* = 0.94.

The polarity is also found in other genomic events, such as alternative splicing, disordered region, and the human-mouse non-synonymous evolution rate (dN) (Figure 7D). Gene expression breadth is a measurement of the number of tissues in which a given gene is expressed. Based on the Unigene reference dataset comprised of 45 body sites, we found that fusion genes’ 5′ partners have greater gene expression breadth than their 3′ partners, independent of tissue selectivity or specificity (Figure 8A–8C). Therefore, fusion genes’ 3′ partners are more tissue –specific.

**Figure 9:**
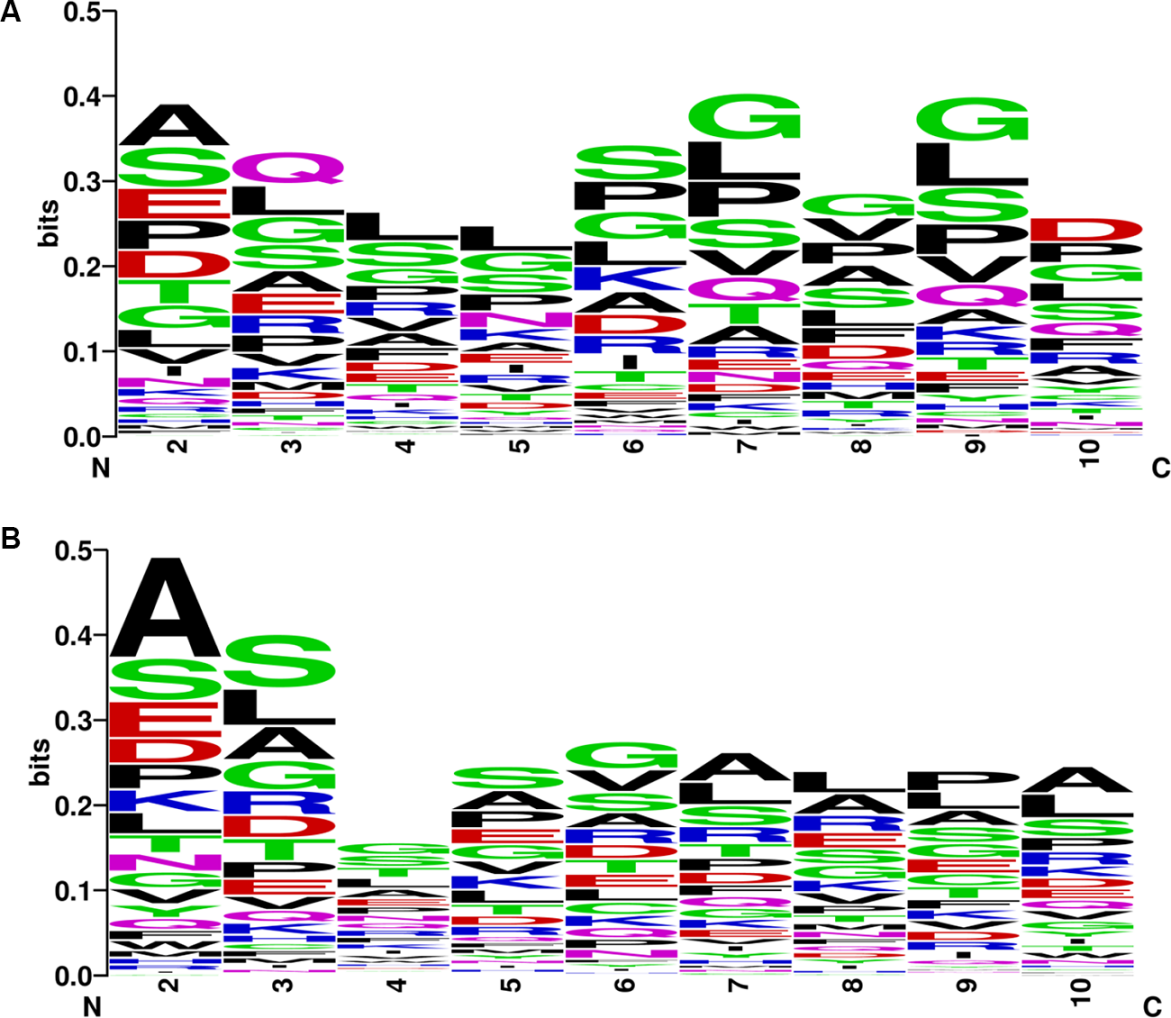
Asymmetric patterns the second to the 10 amino acid of CSGFs (COSMIC driver fusions) 5′ partners (A) and 3′ partners (B) Two matrices based on the frequency of first ten amino acids of the encoded peptides encoded by the 5 and 3 involved genes of CSGFs are plotted with logo diagram respectively. The two diagrams depict distinct patterns of the 5 partners (A) and 3partners.

We further examined the asymmetric pattern at the promoter, 5′ UTR and 3′ UTR of transcripts, as well as encoded protein levels of the CSFGs. Core promoter regions, the 1.5 kb region spanning upstream 1 kb and downstream 0.5 kb from the transcription start site (TSS) of CSGFs (COSMIC driver fusions), show asymmetry in CpG count (Supplementary Figure S2A), TpA count (Supplementary Figure S2B), G4 (GGGG) counts (Supplementary Figure S2C), and C4 (CCCC) counts (Supplementary Figure S2D). The significant sequence features between CSGFs involved 5′ genes and 3′ genes (Two-sample Kolmogorov-Smirnov test *p*-value < 2.2e-16) indicate that 5′ sequence features might be related to the formation of CSGFs. Indeed, 5′ partners in CSGFs show lower overall GC content (Figure 8D, Wilcoxon rank sum test, W = 257,110, *p*-value = 1.95e- 05) but 3′UTR partners in CSGFs doesn't show significant difference in GC content (Figure 8E, Wilcoxon rank sum test with continuity correction, W = 129,190, *p*-value = 0.53) and promoters (Figure 8F, W = 18,896, *p*-value = 0.94). Intriguingly, protein stability related motif of the N-terminal sequences of FG5 and FG3 reveal an asymmetric patterns the second to the 10 amino acid of CSGFs (COSMIC driver fusions) 5′ partners (A) (Figure 9). Since the N-terminal amino acids are crucial protein degradation [44], indicating protein stability might be another critical level of cancer gene fusion functions in turn cancer somatic evolution.

### The connection of somatic copy number alterations and CSGFs

**Figure 10:**
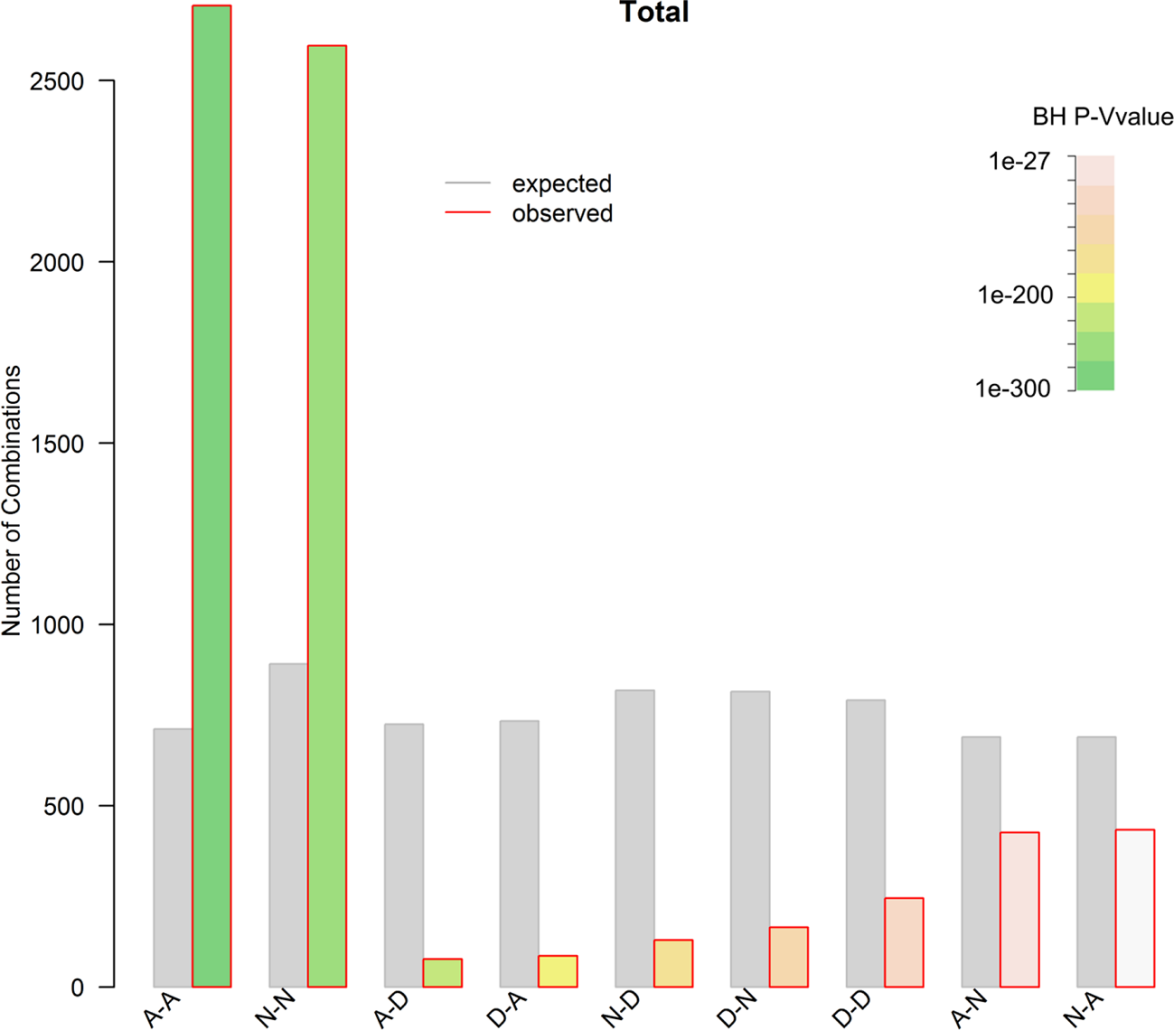
The relationship between CSGFs and SCNAs Both the CSGFs and SCNAs were derived from the TCGA data. To extract a set of high confidence CNVs, a threshold of 0.2 in segment mean value for amplifications and −0.2 for deletions were employed. The observed and expected nine types of combinations (A stands for amplification; N, normal; D, deletion) were plotted. Chi-squared (*X*^2^) test, *X*^2^ = 4375, df = 8, *P* < 2.2e–16. The amplification-amplification (A−A) type is significantly enriched (around 3.8 fold ovepresentation, *P* = 0, binomial test). Meanwhile, both the amplification-deletion (A−D) and deletion-amplification (D−A) types are significantly underrepresented with BH adjusted *P* = 7.22e–222 and *P* = 7.98e-217, respectively.

Somatic copy number alterations (SCNAs) play critical roles in activating oncogenes and inactivating tumour suppressors via affecting a larger fraction of the genome in cancers than any other type of somatic genetic alterations [45–47]. However, little is known about the connection between SCNAs and CSGFs. Here we intersect the SCNAs and CSGFs at individual primary tumour sample level of the 13 type of cancers in TCGA. Intriguingly, among the nine potential combinations, the amplification-amplification (A−A) type is significantly enriched (Figure 10, around 3.8-fold over-representation, *P* = 0, binomial test. Details are presented in Supplementary Table S1). However, the amplification-deletion (A−D) and deletion-amplification (D−A) types are significantly under-represented with 0.11 (BH adjusted *P* = 7.2e-222) and 0.12 (BH adjusted *P* = 8e-217) fold of the expectations, respectively (Figure 10). The result is detailed in Supplementary Table S1. The finding suggests that CSGFs are dynamically evolved in amplification. Hence, we conclude that a large number of CSGFs with amplification are selected during somatic evolution of tumours.

### Functional exploration of CSGFs and CSGFs involved genes

We further examined the asymmetric patterns of functional combinations in CSGFs. We interrogated the TCGA 13-cancer data sets with known kinase (KI) genes [48] and transcription factors (TF) [49]. As shown in the Supplementary Table S2, there are a substantial number of combinations, yet the TF-KI combination is more enriched that is KI-TF (with 3.3- and 2.1-fold enrichment, respectively, and adjusted BH *p* values 5.5e-22 and 3.3e-7, successively). Since most kinases are related to protoncogene function while TFs are enriched in tumor suppressors, it is most likely that 5′ partners have higher propensity to be tumour suppressor while 3′ partners have a larger likelihood to be proto-oncogene. Intriguingly, we further examined the asymmetric pattern of FG5 and FG3 at other levels, including intrinsic protein disorder and cancer signalling pathways. As shown in Supplementary Figure S3, the F5Gs (cancer somatic gene fusions involved 5′ genes) have a higher gene expression breadth and higher intrinsic disorder region score while the F5Gs have more transcripts and longer gene length. Meanwhile F5Gs and F3Gs (cancer somatic gene fusions involved 3′ genes) were asymmetrically mapped to the key cancer gene pathways (Supplementary Figure S4). Thus, it appears to be a functional stratification of CSGFs in terms of F5Gs and F3Gs.

Given that most cancer deaths are due to the development of metastases [50, 51], we examined whether CSGFs involved genes are enriched metastasis genes. Based on PubMed search and collected gene settings, we curated a tumour metastasis gene set [52, 53]. As shown in Supplementary Figure S7, there is a 3.7-fold enrichment of the metastasis genes in the fusion genes setting collected in the COSMIC database (*P* < 5.4e-20, hypergeometric test). The 63 CSGFs involved genes are also listed in the Supplementary Table S3. It appears that CSGFs may have critical role during tumour metastasis.

### Network properties of CSGF involved genes

Next we explored the network properties of CSGF involved genes in the human protein interaction networks. The centrality distribution of cancer related genes, including SMG (somatically mutated cancer driver genes), CPG (cancer predisposition genes), GCG (GWAS cancer associated genes), GMG (HGMD cancer genes), and WGG (human genome genes), are shown in Supplementary Figure S5. CSGF involved genes have a similar distribution pattern compared to putative cancer genes (Supplementary Figure S5). Meanwhile, there is an asymmetric pattern of the centrality of 5′ and 3′ partners (Supplementary Figure S6) and the 5′ partners have less degree that their 3′ counterparts (Supplementary Figure S6, Kolmogorov-Smirnov test, *n* = 5155, D^= 0.5, *p*-value < 2.2e-16). It is likely that those physical interacted pairs are susceptible to become gene fusion pairs. Thus, CSGFs favor gene pairs with strong physical interactions, suggesting a mechanism similar to evolution by “tinkering” [54].

## DISCUSSION

Distinct phylogenomic and genomic features identified through evolutionary studies of the emergence of cancer genes have provided mechanistic insights into the complexity of cancer progression in human. However, less is known about CSGFs involved genes at the genome-wide level since most existing studies focus on discovering novel fusions and testing individual molecular mechanisms [2, 40]. In this study, we show that a substantial number of CSGF involved genes arise from vertebrate whole genome duplications while the involved gene families are of bilateral origin. Consistently, conserved protein domain combinations of the cancer somatic fusion genes appear to be vertebrate specific. Our results provide molecular evidence supporting the hypothesis that the earliest cancer occurred in vertebrates [39]. Indeed, the earliest known unequivocal neoplastic case was found on the partial skeleton of a North American lower carboniferous (about 300 million years BP) fossil fish, *Phanerosteon mirabile* [55].

Distinct genomic features of CSGFs may have both theoretical and practical implications. There are clear patterns of the polarity of CSGFs: i) F5Gs appear to be less globally expressed in contrast to a recent study which showed more global expression of cancer genes; ii) the selection signatures of 3′ UTRs of 5′ UTRs are linked to an miRNA regulation network [9, 56]; iii) at the protein level, there are functional selection footprints of N-terminal degradation rules [44]; iv) there are intrinsic disordered signatures at the joint site of CSGFs [56]. Such observed structure and function relationships not only have the potential to enable us to better understand the functions of CSGFs and more accurately predict CSGFs but also implicate novel therapeutics including targeting cancer somatic fused tyrosine kinase (TK) fusions and activating multiple CSGFs simultaneously [12].

DNA breakpoint signatures provide new insights into the mechanisms underlying CSGFs. However, most studies focused upon germline translocation breakpoints, involving four distinct mechanisms including non-homologous end joining (NHEJ), non-allelic homologous recombination (NAHR), transposition, DNA replication mechanisms [40]. For somatic cases, especially for those in carcinomas, arising mechanisms have been largely unknown(26,28). These overrepresented motifs further highlight the key role of inflammation process that might cause genome instability. Given that genome instability can trigger inflammation, there may be a positive feedback loop between two hallmarks of cancer, genome instability and immune disorder [57]. Our study also revealed the connection of CSGFs to promoters, enhancers, H3K4me3, replication time, as well as fragile sites susceptible to rearrangements and translocations. Another important finding from our study is that a significant number of CSGFs are involved in metastasis, the core of cancer mortality.

Taken together, our findings underscore the prerequisites, causes, and consequences of CSGFs and further our knowledge of CSGFs at the evolutionary, structural and functional levels. The striking features uncovered by integration of phylogenomic, functional genomic, protein interaction data have both theoretical and clinical implications for further testing.

## MATERIALS AND METHODS

### CSGF data sets and annotations

CSGFs datasets (Table 1), including the COSMIC [22], Genetech cell line fusions [5], TICdb [4], Cancer landscape fusions [7], Mitelman fusions [23], and a recent curation of the TCGA fusions [6], were collected. Specially, the cancer landscape fusions and COSMIC fusions are focused on driver fusions, while other data settings are mixture of a variety of different somatic fusions found in cancer.

Functional genomic data sets (Table 1) including the reference human genomic ENSEMBL data [24], refined human protein coding genes (general and tissue-specific protein-protein interactions [25], FANTOM expression data [26, 27], GTEX data, the standard gene expression data set [29], Illumina's body map, draft human proteome, gene age curation [14], human gene expression atlas [33], as well as the TCGA data [34, 35], were also collected. Gene expression data including both mRNA level and protein level as well as protein interaction network data were tabulated in Table 1. Evolutionary data including gene and gene family age settings have also been listed.

The TCGA SCNAs data set was downloaded from the TCGA site. For a set of high-quality and robust CNVs extracting, the thresholds of 0.2 and −0.2 for segment mean value were used to determine amplifications and deletions, respectively. For CSGFs breakpoint and TGCA SNCAs overlapping, the Granges Bioconductor class was applied in the hg19 reference genome [58].

### Asymmetry statistics

Statistical comparisons were carried out with a non-parameter model with R functions including Wilcoxon Signed Rank or Kruskal Wallis test. The Bland-Altman plot was employed for asymmetry analysis and signal capture [59]. The two-sample Kolmogorov-Smirnov test was used for statistically significant test with the function ‘ks.test’.

### DNA breakpoint sequence motifs analysis

Pan-cancer breakpoint sequences were downloaded from the TICdb (release 3.3, August 2013, http://www.unav.es/genetica/TICdb/) [4]. Meanwhile, a set of breakpoint sequences was got from literatures [5, 6, 60, 61], and sequences were mapped to the hg19 human genome with GenomicAlignment [34]. Motif analysis library was carried out by using the method described in the literature [62]. The null model of overrepresentation was based on a Fisher's exact test by using the R script.

### Protein-protein interaction networks

Gene-gene connection data was downloaded from HumanNet V.1 [63], a probabilistic functional gene network of 18,714 validated protein-encoding genes of *Homo sapiens* (by NCBI March 2007). HumanNet was constructed by a modified Bayesian integration of 21 types of 'omics' data from multiple organisms, with each data type weighted according to how well it links genes that are known to function together in H. Sapiens. Protein-protein interaction data and tissue specific protein interaction data networks were downloaded from the related web sites [25, 64–70]. The “igraph” package [71] was used to compute network properties including, degree distribution, betweenness centrality, associativity, closeness, short-path, as well as density.

## SUPPLEMENTARY MATERIALS FIGURES AND TABLES





## References

[1] Rowley JD (1973). Letter: A new consistent chromosomal abnormality in chronic myelogenous leukaemia identified by quinacrine fluorescence and Giemsa staining. Nature.

[2] Mertens F, Johansson B, Fioretos T, Mitelman F (2015). The emerging complexity of gene fusions in cancer. Nat Rev Cancer.

[3] Jimeno Yepes A, Verspoor K (2014). Literature mining of genetic variants for curation: quantifying the importance of supplementary material. Database (Oxford).

[4] Novo FJ, de Mendibil IO, Vizmanos JL (2007). TICdb: a collection of gene-mapped translocation breakpoints in cancer. BMC Genomics.

[5] Klijn C, Durinck S, Stawiski EW, Haverty PM, Jiang Z, Liu H, Degenhardt J, Mayba O, Gnad F, Liu J, Pau G, Reeder J, Cao Y (2015). A comprehensive transcriptional portrait of human cancer cell lines. Nat Biotechnol.

[6] Yoshihara K, Wang Q, Torres-Garcia W, Zheng S, Vegesna R, Kim H, Verhaak RG (2014). The landscape and therapeutic relevance of cancer-associated transcript fusions. Oncogene.

[7] Vogelstein B, Papadopoulos N, Velculescu VE, Zhou S, Diaz LA, Kinzler KW (2013). Cancer genome landscapes. Science.

[8] Engreitz JM, Agarwala V, Mirny LA (2012). Three-dimensional genome architecture influences partner selection for chromosomal translocations in human disease. PLoS One.

[9] Shugay M, Ortiz de Mendibil I, Vizmanos JL, Novo FJ (2012). Genomic hallmarks of genes involved in chromosomal translocations in hematological cancer. PLoS Comput Biol.

[10] Cairns J (1975). Mutation selection and the natural history of cancer. Nature.

[11] Greaves M, Maley CC (2012). Clonal evolution in cancer. Nature.

[12] Liu H, Westergard TD, Cashen A, Piwnica-Worms DR, Kunkle L, Vij R, Pham CG, Dipersio J, Cheng EH, Hsieh JJ (2014). Proteasome Inhibitors Evoke Latent Tumor Suppression Programs in Pro-B MLL Leukemias through MLL-AF4. Cancer Cell.

[13] Zhao Y, Epstein RJ (2008). Programmed genetic instability: a tumor-permissive mechanism for maintaining the evolvability of higher species through methylation-dependent mutation of DNA repair genes in the male germ line. Mol Biol Evol.

[14] Domazet-Loso T, Tautz D (2010). Phylostratigraphic tracking of cancer genes suggests a link to the emergence of multicellularity in metazoa. BMC Biol.

[15] Yao F, Kausalya JP, Sia YY, Teo AS, Lee WH, Ong AG, Zhang Z, Tan JH, Li G, Bertrand D, Liu X, Poh HM, Guan P (2015). Recurrent Fusion Genes in Gastric Cancer: CLDN18-ARHGAP26 Induces Loss of Epithelial Integrity. Cell Rep.

[16] van Heesch S, Simonis M, van Roosmalen MJ, Pillalamarri V, Brand H, Kuijk EW, de Luca KL, Lansu N, Braat AK, Menelaou A, Hao W, Korving J, Snijder S (2014). Genomic and functional overlap between somatic and germline chromosomal rearrangements. Cell Rep.

[17] Zheng S, Fu J, Vegesna R, Mao Y, Heathcock LE, Torres-Garcia W, Ezhilarasan R, Wang S, McKenna A, Chin L, Brennan CW, Yung WK, Weinstein JN (2013). A survey of intragenic breakpoints in glioblastoma identifies a distinct subset associated with poor survival. Genes Dev.

[18] Sima J, Gilbert DM (2014). Complex correlations: replication timing and mutational landscapes during cancer and genome evolution. Curr Opin Genet Dev.

[19] Lorenz S, Baroy T, Sun J, Nome T, Vodak D, Bryne JC, Hakelien AM, Fernandez-Cuesta L, Mohlendick B, Rieder H, Szuhai K, Zaikova O, Ahlquist TC (2016). Unscrambling the genomic chaos of osteosarcoma reveals extensive transcript fusion, recurrent rearrangements and frequent novel TP53 aberrations. Oncotarget.

[20] Li Y, Schwab C, Ryan SL, Papaemmanuil E, Robinson HM, Jacobs P, Moorman AV, Dyer S, Borrow J, Griffiths M, Heerema NA, Carroll AJ, Talley P (2014). Constitutional and somatic rearrangement of chromosome 21 in acute lymphoblastic leukaemia. Nature.

[21] Cheng C, Zhou Y, Li H, Xiong T, Li S, Bi Y, Kong P, Wang F, Cui H, Li Y, Fang X, Yan T, Li Y (2016). Whole-Genome Sequencing Reveals Diverse Models of Structural Variations in Esophageal Squamous Cell Carcinoma. Am J Hum Genet.

[22] Forbes SA, Beare D, Gunasekaran P, Leung K, Bindal N, Boutselakis H, Ding M, Bamford S, Cole C, Ward S, Kok CY, Jia M, De T (2015). COSMIC: exploring the world's knowledge of somatic mutations in human cancer. Nucleic Acids Res.

[23] Mitelman F, Johansson B, Mertens F (2007). The impact of translocations and gene fusions on cancer causation. Nat Rev Cancer.

[24] Flicek P, Amode MR, Barrell D, Beal K, Billis K, Brent S, Carvalho-Silva D, Clapham P, Coates G, Fitzgerald S, Gil L, Giron CG, Gordon L (2014). Ensembl. Nucleic Acids Res.

[25] Barshir R, Shwartz O, Smoly IY, Yeger-Lotem E (2014). Comparative analysis of human tissue interactomes reveals factors leading to tissue-specific manifestation of hereditary diseases. PLoS Comput Biol.

[26] Motakis E, Guhl S, Ishizu Y, Itoh M, Kawaji H, de Hoon M, Lassmann T, Carninci P, Hayashizaki Y, Zuberbier T, Forrest AR, Babina M, consortium F (2014). Redefinition of the human mast cell transcriptome by deep-CAGE sequencing. Blood.

[27] Andersson R, Gebhard C, Miguel-Escalada I, Hoof I, Bornholdt J, Boyd M, Chen Y, Zhao X, Schmidl C, Suzuki T, Ntini E, Arner E, Valen E (2014). An atlas of active enhancers across human cell types and tissues. Nature.

[28] Mele M, Ferreira PG, Reverter F, DeLuca DS, Monlong J, Sammeth M, Young TR, Goldmann JM, Pervouchine DD, Sullivan TJ, Johnson R, Segre AV, Djebali S (2015). Human genomics. The human transcriptome across tissues and individuals. Science.

[29] Su AI, Wiltshire T, Batalov S, Lapp H, Ching KA, Block D, Zhang J, Soden R, Hayakawa M, Kreiman G, Cooke MP, Walker JR, Hogenesch JB (2004). A gene atlas of the mouse and human protein-encoding transcriptomes. Proc Natl Acad Sci U S A.

[30] Florea L, Song L, Salzberg SL (2013). Thousands of exon skipping events differentiate among splicing patterns in sixteen human tissues. F1000Res.

[31] Wilhelm M, Schlegl J, Hahne H, Moghaddas Gholami A, Lieberenz M, Savitski MM, Ziegler E, Butzmann L, Gessulat S, Marx H, Mathieson T, Lemeer S, Schnatbaum K (2014). Mass-spectrometry-based draft of the human proteome. Nature.

[32] Kim MS, Pinto SM, Getnet D, Nirujogi RS, Manda SS, Chaerkady R, Madugundu AK, Kelkar DS, Isserlin R, Jain S, Thomas JK, Muthusamy B, Leal-Rojas P (2014). A draft map of the human proteome. Nature.

[33] Lukk M, Kapushesky M, Nikkila J, Parkinson H, Goncalves A, Huber W, Ukkonen E, Brazma A (2010). A global map of human gene expression. Nat Biotechnol.

[34] Roberts SA, Lawrence MS, Klimczak LJ, Grimm SA, Fargo D, Stojanov P, Kiezun A, Kryukov GV, Carter SL, Saksena G, Harris S, Shah RR, Resnick MA (2013). An APOBEC cytidine deaminase mutagenesis pattern is widespread in human cancers. Nat Genet.

[35] Kandoth C, McLellan MD, Vandin F, Ye K, Niu B, Lu C, Xie M, Zhang Q, McMichael JF, Wyczalkowski MA, Leiserson MD, Miller CA, Welch JS (2013). Mutational landscape and significance across 12 major cancer types. Nature.

[36] Lawrence MS, Stojanov P, Polak P, Kryukov GV, Cibulskis K, Sivachenko A, Carter SL, Stewart C, Mermel CH, Roberts SA, Kiezun A, Hammerman PS, McKenna A (2013). Mutational heterogeneity in cancer and the search for new cancer-associated genes. Nature.

[37] Capra JA, Williams AG, Pollard KS (2012). ProteinHistorian: tools for the comparative analysis of eukaryote protein origin. PLoS Comput Biol.

[38] Ezkurdia I, Juan D, Rodriguez JM, Frankish A, Diekhans M, Harrow J, Vazquez J, Valencia A, Tress ML (2014). Multiple evidence strands suggest that there may be as few as 19 000 human protein-coding genes. Hum Mol Genet.

[39] Capasso LL (2005). Antiquity of cancer. Int J Cancer.

[40] Helleday T, Eshtad S, Nik-Zainal S (2014). Mechanisms underlying mutational signatures in human cancers. Nat Rev Genet.

[41] Ginno PA, Lim YW, Lott PL, Korf I, Chedin F (2013). GC skew at the 5′ and 3′ ends of human genes links R-loop formation to epigenetic regulation and transcription termination. Genome Res.

[42] Gan W, Guan Z, Liu J, Gui T, Shen K, Manley JL, Li X (2011). R-loop-mediated genomic instability is caused by impairment of replication fork progression. Genes Dev.

[43] Stirling PC, Chan YA, Minaker SW, Aristizabal MJ, Barrett I, Sipahimalani P, Kobor MS, Hieter P (2012). R-loop-mediated genome instability in mRNA cleavage and polyadenylation mutants. Genes Dev.

[44] Kim HK, Kim RR, Oh JH, Cho H, Varshavsky A, Hwang CS (2014). The N-terminal methionine of cellular proteins as a degradation signal. Cell.

[45] Zack TI, Schumacher SE, Carter SL, Cherniack AD, Saksena G, Tabak B, Lawrence MS, Zhsng CZ, Wala J, Mermel CH, Sougnez C, Gabriel SB, Hernandez B (2013). Pan-cancer patterns of somatic copy number alteration. Nat Genet.

[46] Beroukhim R, Mermel CH, Porter D, Wei G, Raychaudhuri S, Donovan J, Barretina J, Boehm JS, Dobson J, Urashima M, Mc Henry KT, Pinchback RM, Ligon AH (2010). The landscape of somatic copy-number alteration across human cancers. Nature.

[47] Kim TM, Xi R, Luquette LJ, Park RW, Johnson MD, Park PJ (2013). Functional genomic analysis of chromosomal aberrations in a compendium of 8000 cancer genomes. Genome Res.

[48] Creixell P, Palmeri A, Miller CJ, Lou HJ, Santini CC, Nielsen M, Turk BE, Linding R (2015). Unmasking determinants of specificity in the human kinome. Cell.

[49] Schaefer U, Schmeier S, Bajic VB (2011). TcoF-DB: dragon database for human transcription co-factors and transcription factor interacting proteins. Nucleic Acids Res.

[50] Valastyan S, Weinberg RA (2011). Tumor metastasis: molecular insights and evolving paradigms. Cell.

[51] Eccles SA, Welch DR (2007). Metastasis: recent discoveries and novel treatment strategies. Lancet.

[52] Zhao M, Li Z, Qu H (2015). An evidence-based knowledgebase of metastasis suppressors to identify key pathways relevant to cancer metastasis. Sci Rep.

[53] Sethi N, Kang Y (2011). Unravelling the complexity of metastasis - molecular understanding and targeted therapies. Nat Rev Cancer.

[54] Jacob F (1977). Evolution and tinkering. Science.

[55] Moodie RL (1927). Tumors in the Lower Carboniferous. Science.

[56] Hegyi H, Buday L, Tompa P (2009). Intrinsic structural disorder confers cellular viability on oncogenic fusion proteins. PLoS Comput Biol.

[57] Hanahan D, Weinberg RA (2011). Hallmarks of cancer: the next generation. Cell.

[58] Lawrence M, Huber W, Pages H, Aboyoun P, Carlson M, Gentleman R, Morgan MT, Carey VJ (2013). Software for computing and annotating genomic ranges. PLoS Comput Biol.

[59] Bland JM, Altman DG (1986). Statistical methods for assessing agreement between two methods of clinical measurement. Lancet.

[60] Aguado C, Gaya-Vidal M, Villatoro S, Oliva M, Izquierdo D, Giner-Delgado C, Montalvo V, Garcia-Gonzalez J, Martinez-Fundichely A, Capilla L, Ruiz-Herrera A, Estivill X, Puig M (2014). Validation and genotyping of multiple human polymorphic inversions mediated by inverted repeats reveals a high degree of recurrence. PLoS Genet.

[61] Giacomini CP, Sun S, Varma S, Shain AH, Giacomini MM, Balagtas J, Sweeney RT, Lai E, Del Vecchio CA, Forster AD, Clarke N, Montgomery KD, Zhu S (2013). Breakpoint analysis of transcriptional and genomic profiles uncovers novel gene fusions spanning multiple human cancer types. PLoS Genet.

[62] Stephens PJ, McBride DJ, Lin ML, Varela I, Pleasance ED, Simpson JT, Stebbings LA, Leroy C, Edkins S, Mudie LJ, Greenman CD, Jia M, Latimer C (2009). Complex landscapes of somatic rearrangement in human breast cancer genomes. Nature.

[63] Lee I, Blom UM, Wang PI, Shim JE, Marcotte EM (2011). Prioritizing candidate disease genes by network-based boosting of genome-wide association data. Genome Res.

[64] Juettemann T, Gerloff DL (2011). BISC: binary subcomplexes in proteins database. Nucleic Acids Res.

[65] Motono C, Nakata J, Koike R, Shimizu K, Shirota M, Amemiya T, Tomii K, Nagano N, Sakaya N, Misoo K, Sato M, Kidera A, Hiroaki H (2011). SAHG, a comprehensive database of predicted structures of all human proteins. Nucleic Acids Res.

[66] Chen XW, Jeong JC, Dermyer P (2011). KUPS: constructing datasets of interacting and non-interacting protein pairs with associated attributions. Nucleic Acids Res.

[67] Keshava Prasad TS, Goel R, Kandasamy K, Keerthikumar S, Kumar S, Mathivanan S, Telikicherla D, Raju R, Shafreen B, Venugopal A, Balakrishnan L, Marimuthu A, Banerjee S (2009). Human Protein Reference Database—2009 update. Nucleic Acids Res.

[68] Yang Y, Boss IW, McIntyre LM, Renne R (2014). A systems biology approach identified different regulatory networks targeted by KSHV miR-K12-11 in B cells and endothelial cells. BMC Genomics.

[69] Sinha A, Nagarajaram HA (2014). Nodes occupying central positions in human tissue specific PPI networks are enriched with many splice variants. Proteomics.

[70] Vogt I, Prinz J, Worf K, Campillos M (2014). Systematic analysis of gene properties influencing organ system phenotypes in mammalian perturbations. Bioinformatics.

[71] Csardi G NT (2006). The igraph software package for complex network research. InterJournal, Complex Systems.

