# Supplementary material for: Distinct distributions of genomic features of the 5’ and 3’ partners of coding somatic cancer gene fusions: arising mechanisms and functional implications
Source: Oncotarget. 2016 Jul 20;8(40):66769–83. doi: 10.18632/oncotarget.10734 (PMC5620135; doi:10.18632/oncotarget.10734)
Supplement: Supplementary file 1 [file oncotarget-08-66769-s001.pdf]

Distinct distributions of genomic features of the 5′ and 3′ partners of coding somatic cancer gene fusions: arising mechanisms and functional implications

Supplementary Materials

Supplementary Table S1: Enrichment of SCNAs in CSGFs

Combination	Expected	Observed	Fold (O/E)#	Adjusted.P.value (BH)##
A-A	711	2707	3.81	0
N-N	891	2596	2.91	0
N-A	689	433	0.63	6.16E-28
A-N	689	426	0.62	1.83E-29
D-D	791	245	0.31	1.85E-124
D-N	814	164	0.20	8.88E-185
N-D	818	129	0.16	6.30E-214
D-A	733	86	0.12	9.98E-217
A-D	724	77	0.11	7.22E-222

Fold (O/E), O, observed, E, expected. A: amplification, N: neutral, D: deletion.
Fold > 1, overrepresented; Fold < 1, underrepresented.
Adjusted.P.value BH method adjusted for binomial test.

Supplementary Table S2: Distinct distributions of kinase and transcription factor combinations in the TCGA 13-cancer data set

Combination	Expected	Observed	Fold (O/E)#	Adjusted.P.value (BH)##
KI-KI	5	19	3.80	3.13E-06
TF-KI	31	98	3.27	5.49E-22
KI-OE	178	387	2.19	2.82E-42
KI-TF	31	63	2.10	3.27E-07
TF-TF	178	300	1.69	7.26E-17
TF-OE	1036	1452	1.40	1.26E-38
OE-KI	178	238	1.34	1.28E-05
OE-OE	6022	5256	0.87	8.23E-67
OE-TF	1036	882	0.85	3.00E-07

#Fold (O/E), O, observed, E, expected. KI: kinase, TF: transcription factor, OE: other else.
Fold > 1, overrepresented; Fold < 1, underrepresented.
##Adjusted.*P*.value BH method adjusted for binomial test.

Supplementary Table S3: Fusion genes involved in metastasis. See Supplementary_Table_S3

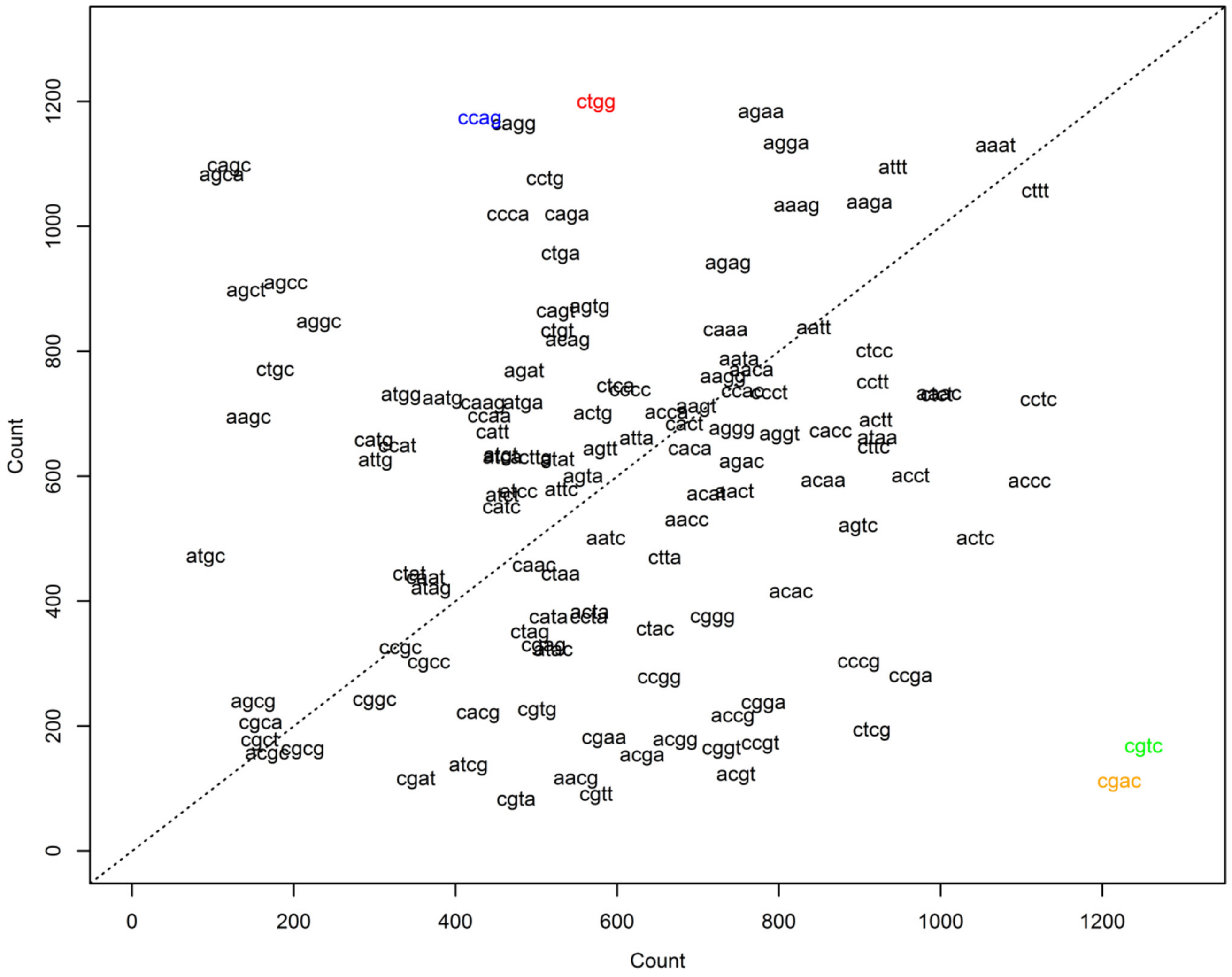

Supplementary Figure S1: Sequence motifs of CSGF sequence breakpoints. The plot illustrates the noted sequence and its complement sequence count, with special signatures indicated by the distinct colour of bases. The analysis shows the asymmetry of the breakpoint sequences from TICdb3.3.

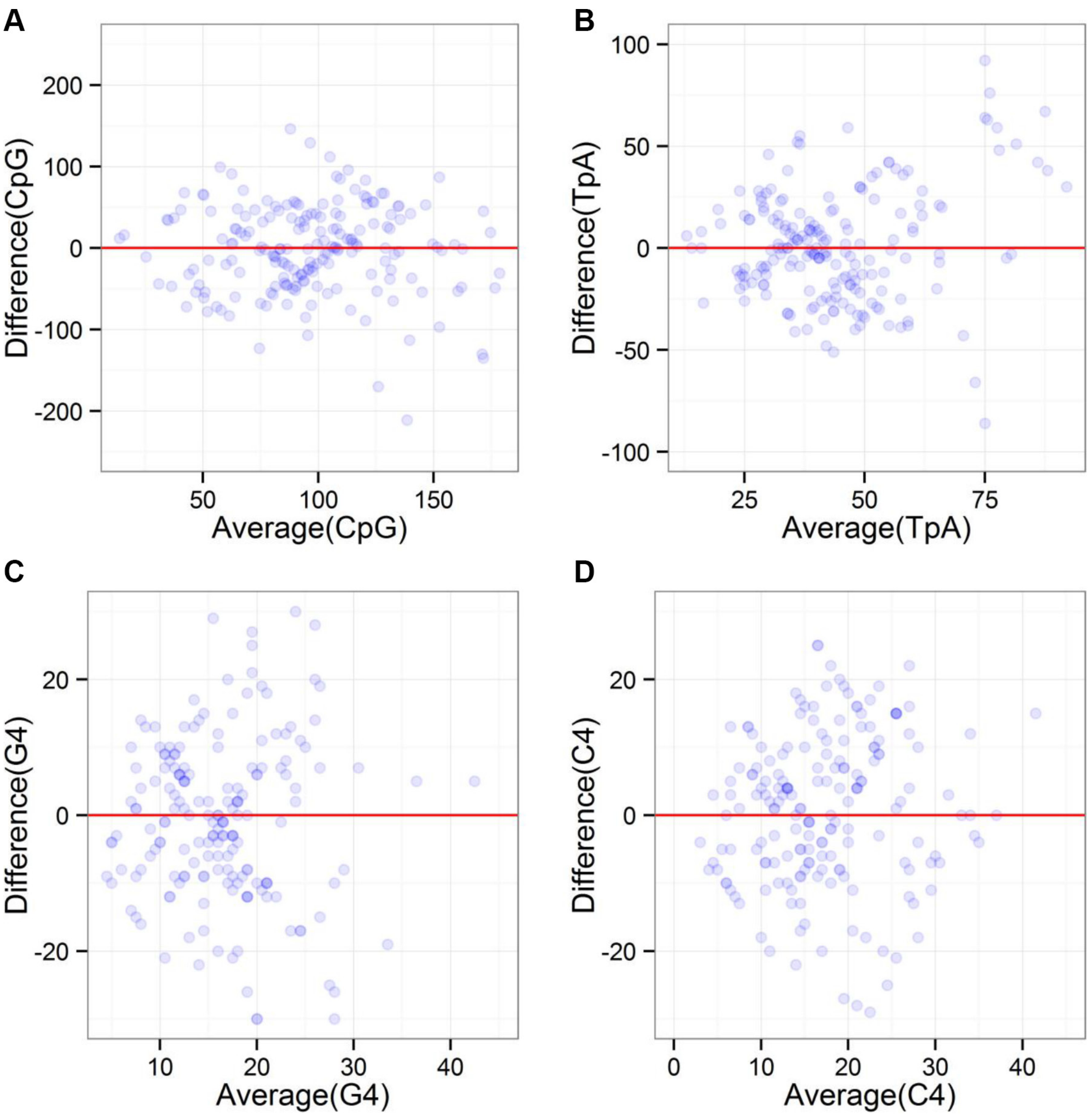

Supplementary Figure S2: The asymmetric patterns of CSGFs (COSMIC driver fusions) in the gene promoter 1.5 kb region spanning upsteam 1kb and downstream 0.5 kb from the transcription start site. (**A**) CpG count comparison between the 5′ fusion partner and the 3′ fusion partner (Kolmogorov-Smirnov test, D = 0.77, *p*-value < 2.2e-16). (**B**) TpA count comparison between the 5′ fusion partner and the 3′ fusion partner. (Kolmogorov-Smirnov test, D = 0.77, *p*-value < 2.2e-16). (**C**) G4 (GGGG) count comparison between the 5′fusion partner and the 3′ fusion partner (D = 0.71, *p*-value < 2.2e-16). D. C4(CCCC) count comparison between the 5′ fusion partner and the 3′ fusion partner (Kolmogorov-Smirnov test, D = 0.63, *p*-value < 2.2e-16).

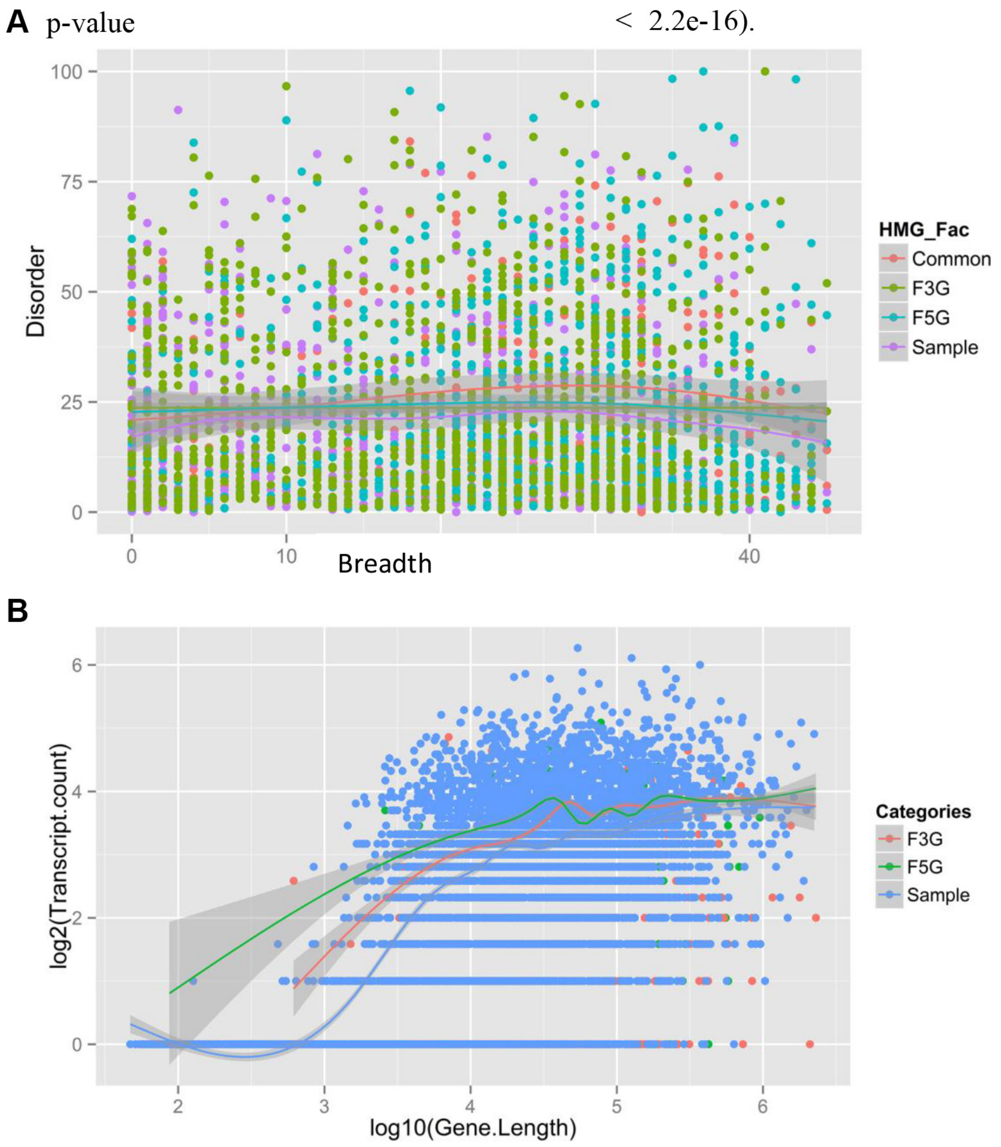

Supplementary Figure S3: Protein disorder and gene length of CSGFs. (**A**) the F5Gs (cancer somatic gene fusions involved 5′ genes) have a higher gene expression breadth and higher intrinsic disorder region score. (**B**) The F5Gs have more transcripts and longer gene length. F5G, cancer somatic gene fusions involved 5′ genes, F3G, cancer somatic gene fusions involved 3′ genes, Sample, whole genome level.

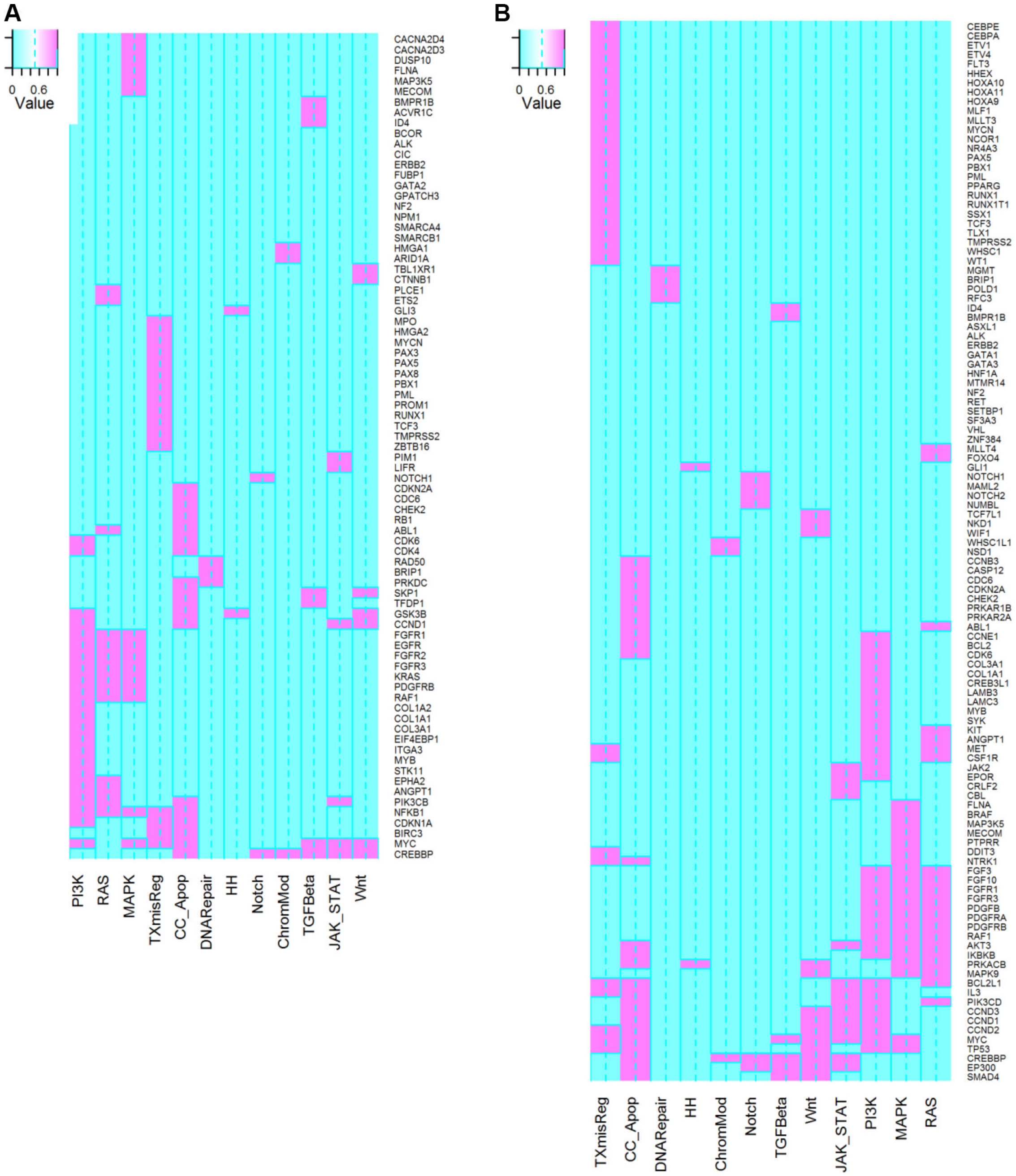

Supplementary Figure S4: The impacts of CSGFs on key cancer progression related molecular pathways. Fusion genes were mapped to the twelve cancer pathways. A pixel in pink color indicates that a gene on the y-axis belongs to a pathway on the x-axis while a pixel in turquoise means a gene doesn't belong to a given pathway. F5Gs and F3Gs were asymmetrically mapped to the key cancer gene pathways. (**A**) FG5 genes. (**B**) FG3 genes.

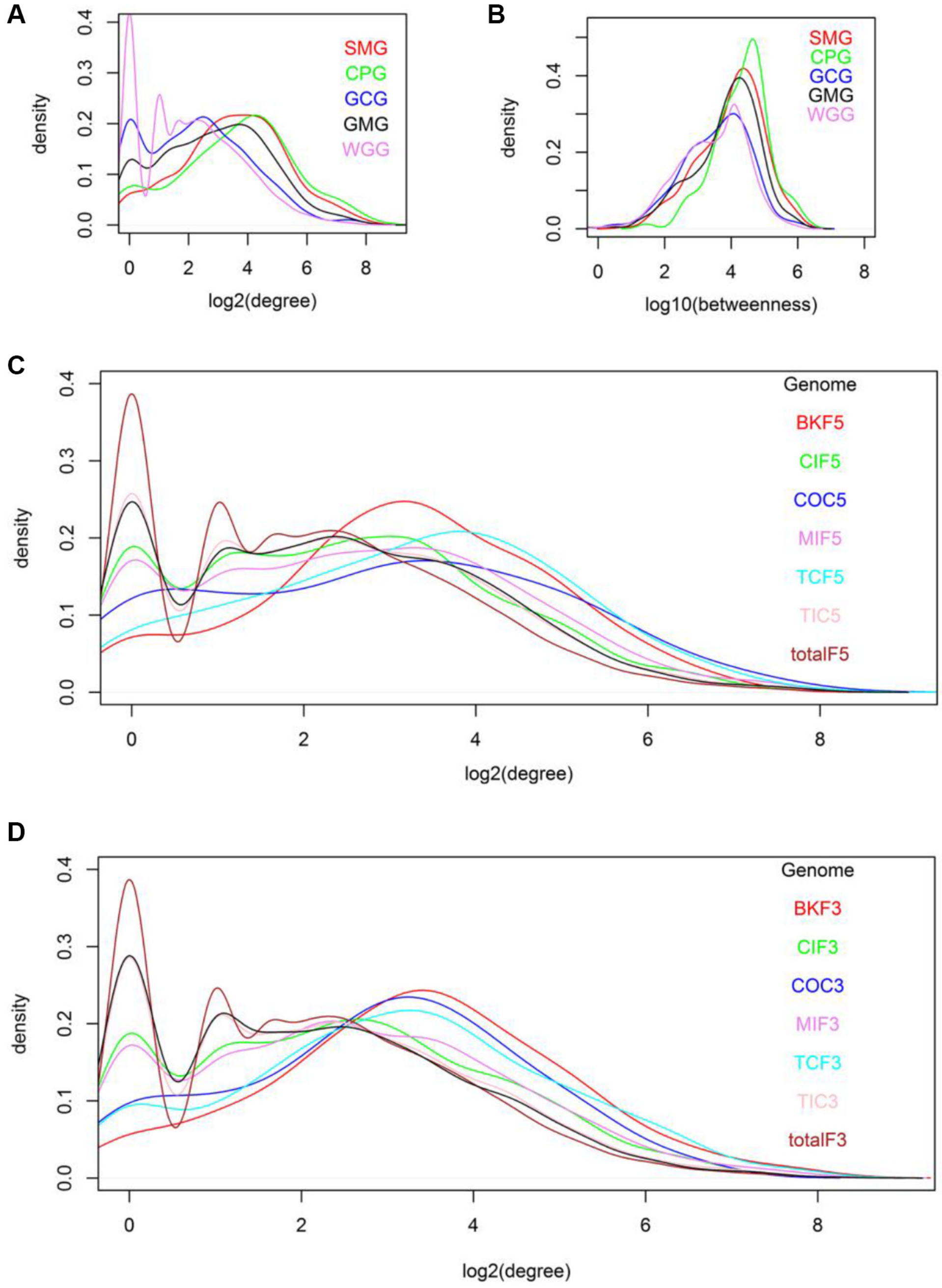

Supplementary Figure S5: Network centrality features of CSGF involved genes. (**A**) The degree distribution of cancer related genes, including SMG (somatically mutated cancer driver genes), CPG (cancer predisposition genes), GCG (GWAS cancer associated genes), GMG (HGMD cancer genes), and WGG (human genome genes). (**B**) The betweenness distribution of cancer related genes. (**C**) The centrality (degree) properties of 5′ partners and 3′ partners of CSGFs as listed in Table 1. Putative driver CSGF sets including BKF and COC. (**D**) The centrality (degree) properties of 3′ partners of CSGFs as listed in Table 1.

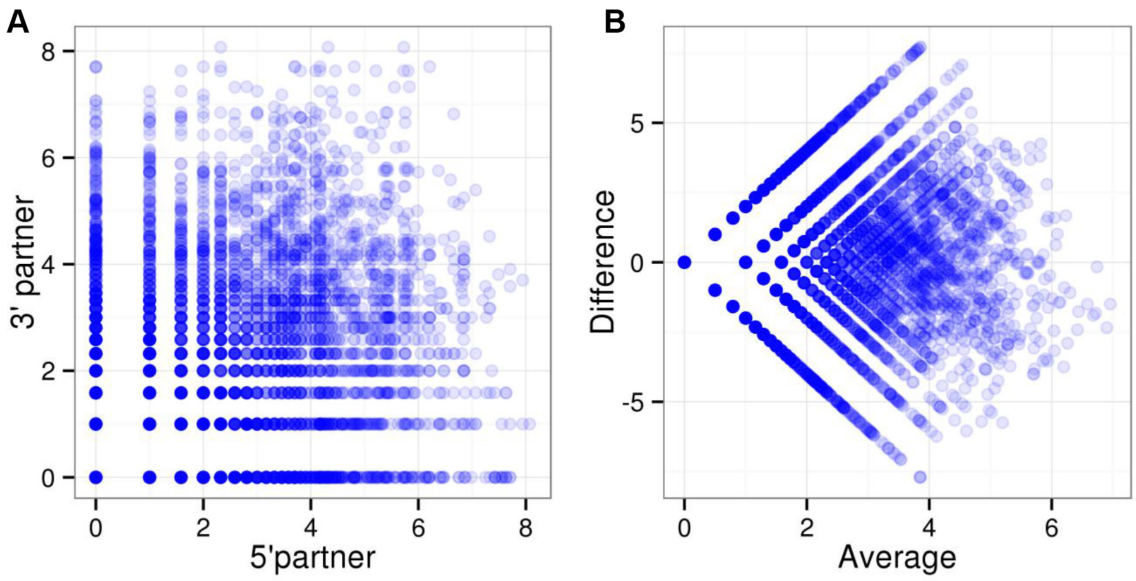

Supplementary Figure S6: The asymmetric pattern of centrality of CSGFs involved genes. (**A**) A degree plot for the cancer somatic fusion gene pairs. (**B**) A Bland-Altman plot for the comparison of 5′ partner and 3′ partner genes. Difference = degree of 5′ partner genes-degree of 3′ partners genes, Average = 1/2(5′ partner genes +3′ partner genes). The 5′ partner genes are less central than their 3' counterparts in the protein-protein interaction network (two-sample Kolmogorov-Smirnov test, $n = 5155$, $D = 0.50$, p-value $< 2.2e-16$).

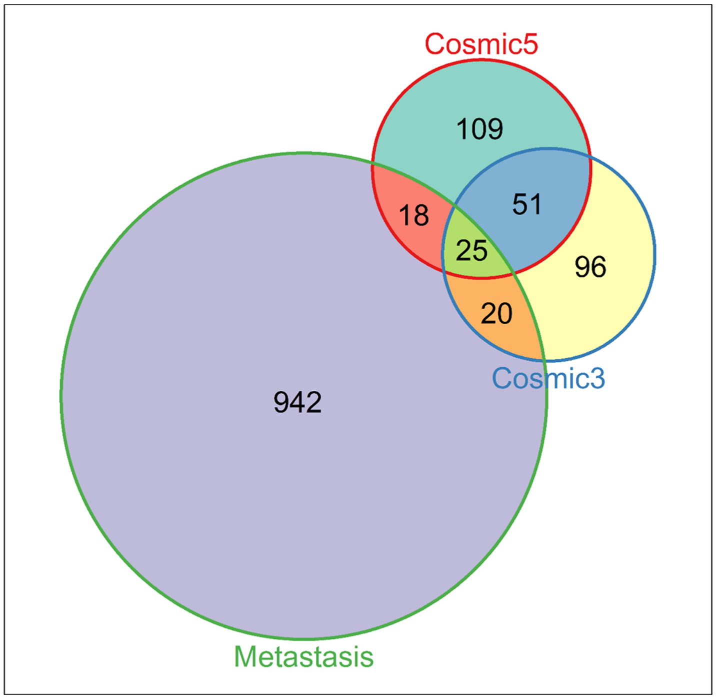

Supplementary Figure S7: The enrichment of metastasis genes in CSGF involved genes. There is a 3.7-fold enrichment of the metastasis genes in the fusion genes setting collected in the COSMIC database ($P < 5.4e-20$, hypergeometric test). The 63 CSGFs involved metastasis genes were listed in the Supplementary Table S3.
