# Supplementary material for: Distinct distributions of genomic features of the 5’ and 3’ partners of coding somatic cancer gene fusions: arising mechanisms and functional implications
Source: Oncotarget. 2016 Jul 20;8(40):66769–83. doi: 10.18632/oncotarget.10734 (PMC5620135; doi:10.18632/oncotarget.10734)
Supplement: Supplementary file 2 [file oncotarget-08-66769-s002.docx]

Supplementary Table S3: Fusion genes involved in metastasis

| hgnc_id | symbol | name | location |
| --- | --- | --- | --- |
| HGNC:76 | ABL1 | ABL proto-oncogene 1, non-receptor tyrosine kinase | 9q34.1 |
| HGNC:115 | ACLY | ATP citrate lyase | 17q21.2 |
| HGNC:132 | ACTB | actin, beta | 7p22 |
| HGNC:427 | ALK | anaplastic lymphoma receptor tyrosine kinase | 2p23 |
| HGNC:1097 | BRAF | B-Raf proto-oncogene, serine/threonine kinase | 7q34 |
| HGNC:1541 | CBL | Cbl proto-oncogene | 11q23.3 |
| HGNC:1750 | CDH11 | cadherin 11 | 16q21 |
| HGNC:13164 | CNBP | CCHC-type zinc finger nucleic acid binding protein | 3q21 |
| HGNC:2197 | COL1A1 | collagen type I alpha 1 | 17q21.33 |
| HGNC:2198 | COL1A2 | collagen type I alpha 2 | 7q21.3 |
| HGNC:2345 | CREB1 | cAMP responsive element binding protein 1 | 2q34 |
| HGNC:18856 | CREB3L1 | cAMP responsive element binding protein 3-like 1 | 11q11 |
| HGNC:2348 | CREBBP | CREB binding protein | 16p13.3 |
| HGNC:2514 | CTNNB1 | catenin beta 1 | 3p21 |
| HGNC:17294 | DAB2IP | DAB2 interacting protein | 9q33.1-q33.3 |
| HGNC:5270 | DNAJB1 | DnaJ heat shock protein family (Hsp40) member B1 | 19p13.12 |
| HGNC:3373 | EP300 | E1A binding protein p300 | 22q13.2 |
| HGNC:3493 | ETV4 | ETS variant 4 | 17q21.31 |
| HGNC:3495 | ETV6 | ETS variant 6 | 12p13 |
| HGNC:3508 | EWSR1 | EWS RNA binding protein 1 | 22q12.2 |
| HGNC:12691 | EZR | ezrin | 6q25.3 |
| HGNC:3688 | FGFR1 | fibroblast growth factor receptor 1 | 8p11.23-p11.22 |
| HGNC:3701 | FHIT | fragile histidine triad | 3p14.2 |
| HGNC:3778 | FN1 | fibronectin 1 | 2q34 |
| HGNC:3819 | FOXO1 | forkhead box O1 | 13q14.1 |
| HGNC:3821 | FOXO3 | forkhead box O3 | 6q21 |
| HGNC:7139 | FOXO4 | forkhead box O4 | Xq13.1 |
| HGNC:4010 | FUS | FUS RNA binding protein | 16p11.2 |
| HGNC:4317 | GLI1 | GLI family zinc finger 1 | 12q13.3 |
| HGNC:5009 | HMGA2 | high mobility group AT-hook 2 | 12q15 |
| HGNC:6407 | KRAS | KRAS proto-oncogene, GTPase | 12p12.1 |
| HGNC:6597 | LIFR | leukemia inhibitory factor receptor alpha | 5p13-p12 |
| HGNC:7373 | MSN | moesin | Xq11.1 |
| HGNC:7545 | MYB | MYB proto-oncogene, transcription factor | 6q22-q23 |
| HGNC:7671 | NCOA4 | nuclear receptor coactivator 4 | 10q11.2 |
| HGNC:7679 | NDRG1 | N-myc downstream regulated 1 | 8q24 |
| HGNC:7765 | NF1 | neurofibromin 1 | 17q11.2 |
| HGNC:7881 | NOTCH1 | notch 1 | 9q34.3 |
| HGNC:7910 | NPM1 | nucleophosmin (nucleolar phosphoprotein B23, numatrin) | 5q35.1 |
| HGNC:7997 | NRG1 | neuregulin 1 | 8p12 |
| HGNC:8029 | NTN1 | netrin 1 | 17p13.1 |
| HGNC:8031 | NTRK1 | neurotrophic receptor tyrosine kinase 1 | 1q21-q22 |
| HGNC:8032 | NTRK2 | neurotrophic receptor tyrosine kinase 2 | 9q22.1 |
| HGNC:8033 | NTRK3 | neurotrophic receptor tyrosine kinase 3 | 15q24-q25 |
| HGNC:8619 | PAX5 | paired box 5 | 9p13.2 |
| HGNC:8632 | PBX1 | pre-B-cell leukemia homeobox 1 | 1q23.3 |
| HGNC:8800 | PDGFB | platelet derived growth factor subunit B | 22q13.1 |
| HGNC:9236 | PPARG | peroxisome proliferator activated receptor gamma | 3p25 |
| HGNC:9380 | PRKACA | protein kinase cAMP-activated catalytic subunit alpha | 19p13.1 |
| HGNC:9388 | PRKAR1A | protein kinase cAMP-dependent type I regulatory subunit alpha | 17q24.2 |
| HGNC:9401 | PRKCE | protein kinase C epsilon | 2p21 |
| HGNC:9674 | PTPRK | protein tyrosine phosphatase, receptor type K | 6q22.2-q22.3 |
| HGNC:9829 | RAF1 | Raf-1 proto-oncogene, serine/threonine kinase | 3p25 |
| HGNC:9967 | RET | ret proto-oncogene | 10q11.2 |
| HGNC:11368 | STAT6 | signal transducer and activator of transcription 6 | 12q13 |
| HGNC:11524 | TACC3 | transforming acidic coiled-coil containing protein 3 | 4p16.3 |
| HGNC:11641 | TCF7L2 | transcription factor 7 like 2 | 10q25.3 |
| HGNC:11752 | TFE3 | transcription factor binding to IGHM enhancer 3 | Xp11.22 |
| HGNC:11998 | TP53 | tumor protein p53 | 17p13.1 |
| HGNC:12665 | VCL | vinculin | 10q22.1-q23 |
| HGNC:12796 | WT1 | Wilms tumor 1 | 11p13 |
| HGNC:12851 | YWHAE | tyrosine 3-monooxygenase/tryptophan 5-monooxygenase activation protein epsilon | 17p13.3 |
| HGNC:12856 | YY1 | YY1 transcription factor | 14q |
